# Bcl11b controls odorant receptor class choice in mice

**DOI:** 10.1038/s42003-019-0536-x

**Published:** 2019-08-07

**Authors:** Takayuki Enomoto, Hidefumi Nishida, Tetsuo Iwata, Akito Fujita, Kanako Nakayama, Takahiro Kashiwagi, Yasue Hatanaka, Hiro Kondo, Rei Kajitani, Takehiko Itoh, Makoto Ohmoto, Ichiro Matsumoto, Junji Hirota

**Affiliations:** 1 Center for Biological Resources and Informatics, Tokyo Institute of Technology, Yokohama, 226-8501 Japan; 2 Department of Life Science and Technology, Graduate School of Life Science and Technology, Tokyo Institute of Technology, Yokohama, 226-8501 Japan; 3 Monell Chemical Senses Center, Philadelphia, PA 19104 USA

**Keywords:** Olfactory receptors, Molecular neuroscience, Gene regulation, Cell fate and cell lineage

## Abstract

Each olfactory sensory neuron (OSN) expresses a single odorant receptor (OR) gene from the class I or class II repertoire in mice. The mechanisms that regulate OR class choice in OSNs remain unknown. Here, we show that the transcription factor Bcl11b determines the OR class to be expressed in OSNs. Both loss- and gain-of-function analyses demonstrate that class I is a default fate of OSNs and that Bcl11b dictates a class II OR choice by suppressing the effect of the J-element, a class I-OR enhancer. We further demonstrate that OSN-specific genetic manipulations of Bcl11b bias the OR class choice, generating mice with “class I-dominant” and “class II-dominant” noses, which display contrasting innate olfactory behaviors to two distinct aversive odorants. Overall, these findings reveal a unique transcriptional mechanism mediating a binary switch for OR class choice that is crucial to both the anatomical and functional organization of the olfactory system.

## Abstract (web summary)

Enomoto et al. show that a transcription factor Bcl11b overrides a default fate of olfactory sensory neurons, class I, to dictate the class II by suppressing a class I-olfactory receptor (OR) enhancer. This study presents a transcriptional binary switch that chooses the OR class, which in turn functionally organizes the olfactory system in mice.

## Introduction

Olfaction, the sense of smell, is essential for the survival of individuals and species. Odorant receptors (ORs), G protein-coupled receptors with a putative seven-transmembrane domain structure, evolved facing different chemical environments and needs, resulting today in the largest gene family in vertebrates^1,2^. OR genes are classified into two classes; class I and class II, based on the homology of their corresponding amino acid sequences^3,4^. Class I genes, which were first identified in fish and frog^5,6^, have persisted throughout the evolution of most vertebrate taxa. On the other hand, class II genes are specific to terrestrial animals and account for ~90% of the mammalian OR repertoires^7^. It has been presumed that class I OR genes represent default- or prototype ORs and that class II ORs have evolved in the tetrapod lineage due to terrestrial adaptation.

The mouse main olfactory epithelium (MOE) consists of several types of sensory neurons: OR-, trace amine-associated receptor-, guanylyl cyclase-D, and vomeronasal receptor-expressing neurons. Individual OR-expressing OSNs express a single functional allele of a single OR gene from either the class I or class II repertoires giving rise to two distinct OR-expressing OSN populations: class I OR-expressing OSNs (class I OSNs) and class II OR-expressing OSNs (class II OSNs)^8–11^. Most class I OSNs reside in the most dorsal region of the MOE and project their axons to the dorsomedial and anteromedial region of the olfactory bulb (OB). By contrast, class II OSNs are distributed throughout the MOE and project axons to the dorsolateral, anterolateral, and ventral OB^10,12–14^. Several studies have demonstrated that the class-specific anatomical domain organization in the OB correlates with known functional odorants response domains^10,14,15^, supporting the hypothesis that class I OSNs detect hydrophilic odorants and class II OSNs detect hydrophobic odorants^4^. Therefore, OR class choice is critical to both the anatomical and functional organization of the olfactory system. However, the molecular mechanisms that regulate OR class choice remain unknown.

The genomic organization of class I and class II genes is quite different. During mammalian OR evolution, class I genes persisted in a single gene cluster on a single chromosome, whereas class II genes spread out over most chromosomes^16,17^. This enigmatic genomic organization of OR genes has made it difficult to unveil the molecular mechanism underlying the OR class choice. Recent studies provide genetic evidence that OR class choice precedes the singular OR gene choice^10,11^. As a result of mutations in OR coding regions (ΔOR) showing that the OR gene that is coexpressed as a second choice pertains to the same class as the ΔOR^10^, it has been proposed that OSNs are fated to choose either class I or class II enhancers/promoters, and thus that class determination is restricted by lineage.

In this study, we demonstrate that the transcription factor Bcl11b (also known as Ctip2) determines the class of OR gene to be expressed in mouse OSNs. Bcl11b is a zinc finger transcription factor, originally identified as a transcriptional repressor that either directly binds to a GC-rich consensus sequence of target genes and/or interacts with a nucleosome remodeling and deacetylase complex to repress target promoters^18–20^. *Bcl11b* is expressed predominantly in the immune, central nervous, and olfactory systems in mice^21,22^. Previous studies of Bcl11b*-*deficient mice have demonstrated critical functions of Bcl11b in these systems except in the main olfactory system^20^. Here, we describe a series of mouse genetic studies that reveal the determinant function of Bcl11b in OR class choice. Expression analysis shows that Bcl11b is predominantly expressed in class II OSNs. Both loss- and gain-of-function mutations of Bcl11b demonstrate that most OSNs throughout the MOE are capable of expressing class I ORs in the absence of Bcl11b. We also show that the presence of Bcl11b dictates the class II OR choice by suppressing the effect of the J element, a class I OR enhancer^11,23^, indicating class I is a default class of OR to be expressed in OSNs. Further, we generated mice with “class I-dominant” and “class II-dominant” noses by manipulating Bcl11b in OSNs. In “class I-dominant” mice, behavioral responses to class I-odors were strengthened while those to class II-odors were weakened, whereas “class II-dominant” mice showed a weakened response to class I odors, demonstrating that OR class choice perturbations in peripheral OSNs change odor perception in mice.

## Results

### Class-specific expression of Bcl11b in OSNs

In the mouse MOE, OSNs mainly comprise two types, class I- and class II-OSNs. Because cell-type specifications are often controlled by transcription factors, we screened OR class-specific transcription factors using a gene expression database to identify candidate molecules that may specify OR class in OSNs^24,25^. OSNs express OR genes at latest in the immature stage^26^; thus, we focused on 1434 transcription factors that are expressed in immature OSNs. Among them, the expression patterns of highly expressed genes (top 10%) in the MOE were analyzed by in situ hybridization (ISH) and using ISH images of the Gene eXpression Database (GXD)^27^ looking for expression patterns that may reflect the class-specific OSN distributions. We found that only Bcl11b showed such expression pattern, i.e., highly expressed in the ventral MOE, where class II OSNs exclusively reside.

**Fig. 1:**
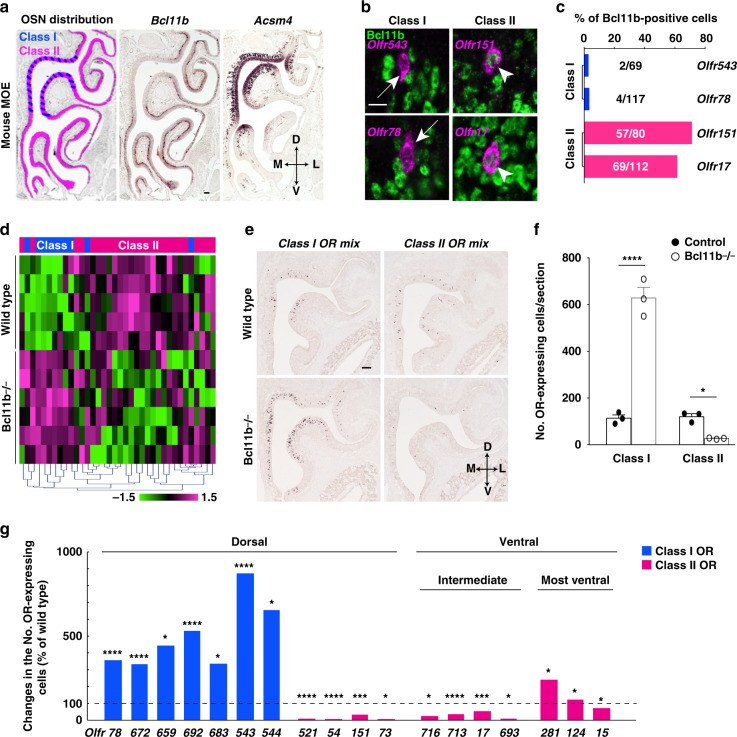
Loss-of-function mutation of Bcl11b biases the OR class choice of OSNs. **a** Distributions of class I (blue) and class II OSNs (magenta) and ISH with RNA probes for *Bcl11b* and a dorsal marker, *Acsm4*, in consecutive coronal sections of the MOE at P30. **b** Combination of IHC for Bcl11b (green) and ISH for OR genes (magenta). Arrowheads and arrow indicate co-labeled and not co-labeled OSNs, respectively. **c** Bar graphs showing the percentages of Bcl11b-positive cells that are labeled with each OR probe (*n* = 3 animals. The quantification data are summarized in Supplementary Data 1). **d** Microarray analysis of the expression of OR genes in the wild type and Bcl11b−/− MOE. A heat-map representation was obtained by hierarchical clustering using 36 OR gene probe sets (blue: class I genes; magenta: class II genes). Each row refers to independent preparations (*n* = 5 control mice, 6 Bcl11b−/− mice). Color scale indicates the log_2_ value of the signal intensity of OR gene normalized to the internal control, *GAPDH* (Supplementary Data 2). **e** ISH with mixed RNA probes for the eight class I and the eight class II genes in coronal sections of the wild type and Bcl11b−/− MOE at P0. **f** Quantification of the number of OSNs expressing class I or class II genes per section (control: black circles, Bcl11b−/−: open circles). Bar represents the mean values ± s.e.m. The quantification data and number of animals analyzed are summarized in Supplementary Data 1. **g** Bar graphs showing the percentage of change in the number of cells expressing each OR gene in Bcl11b−/− versus wild type mice (serial sections throughout the MOE at 100 μm interval were analyzed). The quantification data and number of animals analyzed are summarized in Supplementary Data 1. *p < 0.05, **p < 0.01, ***p < 0.005, ****p < 0.001 (two-tailed *t*-test, *n* = at least 3, as the correction for multiple testing, *p*-values were adjusted based on false-discovery rate using the Benjamini–Hochberg (BH) method). D, dorsal; V, ventral; M, medial; L, lateral. Scale bars, 100 μm in (**a**) and (**e**); 10 μm in (**b**)

The expression of *Bcl11b* has been reported in the embryonic MOE of mice^21^, but its detailed expression patterns have not been characterized. We first analyzed the expression of *Bcl11b* using ISH and Bcl11b by immunohistochemistry (IHC). We observed that *Bcl11b* was expressed in the olfactory system during development up to adulthood, and that expression patterns changed dynamically (Fig. 1a and Supplementary Fig. 1a–c). In a given olfactory neuroepithelium, Bcl11b expression was started between late neuronal precursors and differentiating neurons in the OSN lineage. We observed a consistent variation in labeling intensity with the anti-Bcl11b antibody between cell types, arguing that Bcl11b is strongly expressed early in the OSN lineage and that this expression decreases as OSNs mature (Supplementary Fig. 1d). In addition, *Bcl11b* was strongly expressed in the ventral MOE and weakly in the dorsal MOE (Fig. 1a); this ventral enrichment of *Bcl11b* expression was observed from E14.5 (Supplementary Fig. 1a).

Because most class I genes are expressed exclusively in the dorsal MOE and class II genes are expressed throughout the MOE^10,12–14^, we hypothesized that the expression of class I and class II genes correlate with that of *Bcl11b*. To examine this hypothesis, we analyzed the coexpression of Bcl11b with each class of OR genes. The double-labeling analysis revealed that most OSNs expressing class I genes *Olfr543* and *Olfr78* were Bcl11b*-*negative. In contrast, ~60–70% of OSNs expressing either the dorsal class II gene *Olfr151* or the ventral class II gene *Olfr17* were Bcl11b-positive (Fig. 1b, c and Supplementary Data 1). In the ventral MOE, most immature class II OSNs expressed Bcl11b, and the expression of Bcl11b gradually decreased as OSNs matured (Supplementary Fig. 1d). It is conceivable that the remaining 30–40% of *Olfr151*- and *Olfr17*- expressing OSNs were mature neurons, in which the expression level of Bcl11b was under the detection threshold of our experimental conditions. These results indicate that Bcl11b is expressed predominantly in class II OSNs. The class-specific expression of Bcl11b suggests that Bcl11b plays a critical role in the OR class choice in OSNs.

### Bcl11b-deficiency favors class I OR choice over class II

To elucidate the function of Bcl11b, we conducted loss-of-function analyses using conventional Bcl11b knockout (Bcl11b−/−) mice^28^. Histological analysis showed no overt abnormalities in the gross morphology of the MOE throughout the developmental stages that we analyzed (Supplementary Fig. 2a). Next, we assessed the impact of Bcl11b deficiency on gene expression using DNA microarrays. Clustering analysis of gene expression profiles identified increased and decreased genes in the Bcl11b−/− MOE (Supplementary Fig. 2b and Supplementary Data 2). Interestingly, in the absence of Bcl11b, class I OR mRNA levels were increased, whereas those of class II were decreased along with the markers for mature OSNs (Fig. 1d and Supplementary Fig. 2c–e). Expression levels of immature OSN markers were unchanged in the Bcl11b−/− MOE, and those of mature OSN markers decreased only in the ventral MOE (Supplementary Fig. 2c–e), suggesting that OSNs (at least those located in the dorsal MOE) can differentiate to reach maturity in the absence of Bcl11b. The number of apoptotic cells increased in the ventral MOE of Bcl11b−/− (Supplementary Fig. 2f, g), suggesting that decreased expression of mature OSN markers in the ventral MOE of Bcl11b−/− mice reflect increased apoptosis during differentiation.

To confirm the microarray data, we performed ISH using mixed probes for class I and class II genes. The number of OSNs expressing class I genes was increased in the Bcl11b−/− MOE, whereas that of class II was decreased (Fig. 1e, f). Intriguingly, a broad distribution of class I OSNs was observed throughout the MOE of Bcl11b−/− mice, crossing the border of the dorsally restricted class I region characteristic of wild type mice. The expansion of the expressing region was gene-specific, because some class I-expressing OSNs were distributed throughout the MOE in Bcl11b−/− mice, whereas others were not (Supplementary Fig. 2h). ISH and IHC for the dorsal MOE markers, *Acsm4* and NAD(P)H quinone dehydrogenase 1 (NQO1) demonstrated that these markers were not expressed in the ventral MOE of Bcl11b−/−, suggesting that ectopic class I-OSNs in the ventral MOE of Bcl11b−/− have different molecular characteristics than wild type class I OSNs (Supplementary Fig. 2i). Quantitative analyses showed that the number of each class I OSN was greatly increased in Bcl11b−/− mice relative to their wild type counterparts, whereas those of most class II OSNs were decreased (Fig. 1g and Supplementary Data 1). These results suggest that Bcl11b negatively regulates the expression of class I genes and/or activates the expression of class II genes. However, some exceptions were noted for class II genes: the number of the most ventral class II gene *Olfr281*- and *Olfr124-*expressing OSNs increased. Both *Olfr281* and *Olfr124* are expressed in the septal organ, in which multiple ORs are expressed in single OSNs^29,30^, suggesting that these exceptional class II genes are not regulated like other class II genes.

### Axonal projections and glomerular organization in Bcl11b−/−

**Fig. 2:**
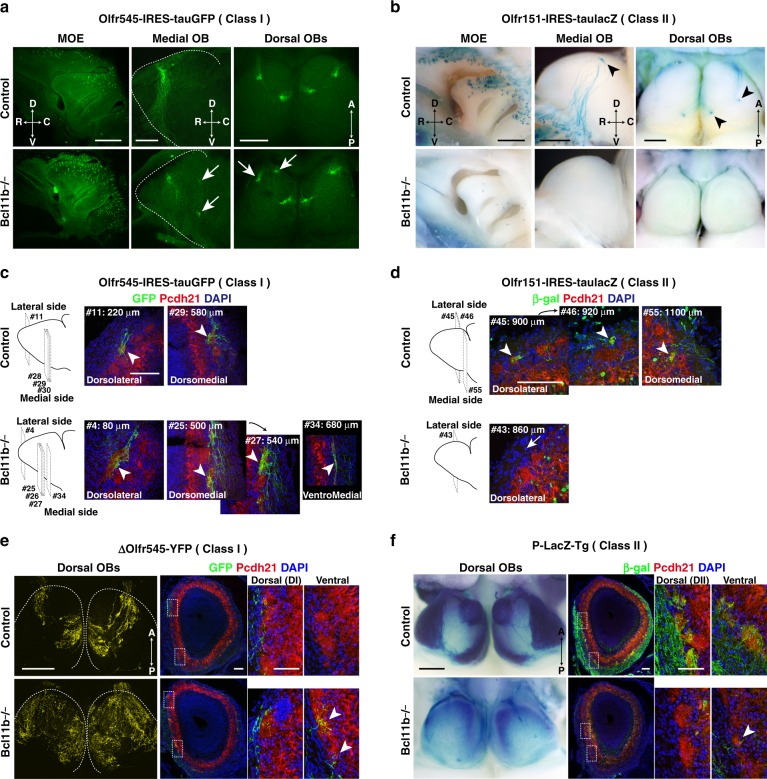
Abnormal axonal projections and glomerular domain organization in Bcl11b−/− mice. **a**–**d** Axonal projections of class I-OSNs and class II-OSNs were visualized using Olfr545-IRES-tauGFP mice (**a**, **c**) and Olfr151-IRES-taulacZ mice (**b**, **d**), respectively with control and Bcl11b−/− backgrounds. **a** Olfr545-expressing class I-OSNs project to ectopic and multiple glomeruli in the Bcl11b−/− OB (arrows). **b** Olfr151-expressing class II OSNs send their axon to a few glomeruli in the OB of control mice (arrowheads), while those of Bcl11b−/− mice are barely detected. **c** Axonal termini of Olfr545-expressing class I-OSNs were immunostained using anti-GFP (green) and anti-protocadherin (Pcdh) 21 (red) antibodies and counterstained with DAPI (blue). Numbers represent the section numbers of Olfr545-positive axonal termini observed in the consecutive coronal sections of OB. Arrowheads indicate Olfr545-positive glomeruli. **d** Axonal termini of Olfr151-expressing class II OSNs were immunostained using anti-β-gal (green) and anti-Pcdh21 (red) antibodies and counterstained with DAPI (blue). The section number of Olfr151-positive axonal termini observed were shown as in (**d**). Arrowheads indicate Olfr151-positive glomeruli. Arrow indicates Olfr151-positive axonal terminus that did not innervate into the glomerular layer. **e** Axonal projection domains of class I OSNs were visualized using ΔOlfr545-YFP mice with control and Bcl11b−/− backgrounds. Dotted lines indicate the OB outline. Coronal sections of the OB were immunostained as in (**c**). Dotted boxes are magnified. Arrowheads indicate ΔOlfr545-positive axonal termini in the ventral OB. **f** Axonal projection domains of class II OSNs in P-lacZ Tg mice with control and Bcl11b−/− backgrounds. Coronal sections of the OB were immunostained as in (**d**). Dotted boxes are magnified. D, dorsal; V, ventral; R, rostral; C, caudal; A, anterior; P, posterior. Scale bars, 100 μm

We next examined the effects of the biased OR class choice on OSN axonal projections and glomerular organization in OB. Bcl11b−/− mice displayed no obvious abnormalities in morphology, cellular composition, or survival in OB and layer organization (Supplementary Fig. 3a–c). To examine the axonal projections of OSNs expressing a given OR, we used gene-targeted strains in which the axons of OSN expressing a given OR allele are labeled by GFP fluorescence or β-galactosidase activity. In Olfr545-IRES-tauGFP mice^10^ bearing the Bcl11b−/− deletion, Olfr545-expressing class I-OSNs were distributed throughout the MOE (Fig. 2a and Supplementary Fig. 2f) and projected their axons to multiple glomeruli in the dorsal OB, as well as to ectopic glomeruli in the ventral OB (Fig. 2a, c). In another class I knockin line (Olfr78-IRES-tauLacZ) bearing the Bcl11b−/−deletion^31^, Olfr78-expressing class I-OSNs were restricted to the dorsal MOE and sent their axons to multiple glomeruli in the dorsal OB (Supplementary Fig. 3d, f). In contrast, neither the axon bundle nor the glomerular formation of Olfr151-expressing class II-OSNs^32^ was observed (Fig. 2b, d). Finally, in a class II knockin line (Olfr17-IRES-tauLacZ)^33^, when lacking Bcl11b, a reduced number of Olfr17-expressing OSNs sent their axons to tiny multiple glomeruli (Supplementary Fig. 3e, g).

We examined the domain organization in the OB using two mouse lines, ΔOlfr545-YFP and P-LacZ Tg, which label class I-OSN and class II-OSN populations, respectively^10^. In ΔOlfr545-YFP mice with control backgrounds (Bcl11b+/+), class I OSN axons selectively innervated the class I domain, a circumscribed region of the dorsal-medial and anterior-medial OB. In Bcl11b−/− mice, the axonal projection domain was greatly enlarged and covered almost the whole dorsal area of the OB. IHC showed that class I OSN axons innervated both dorsal and ventral glomeruli in Bcl11b−/− OBs (Fig. 2e). By introducing the Bcl11b null mutation into P-LacZ Tg mice, the β-gal+ class II OSN projection domain almost disappeared in the dorsal OB. IHC demonstrated that class II glomeruli were barely detectable in the Bcl11b−/− OB, and few axonal innervations were observed in the most ventral Bcl11b−/− OBs (Fig. 2f). Thus, loss-of-function of Bcl11b disrupts the glomerular domain organization in the OB.

### OSN-specific depletion of Bcl11b switches the OR class

**Fig. 3:**
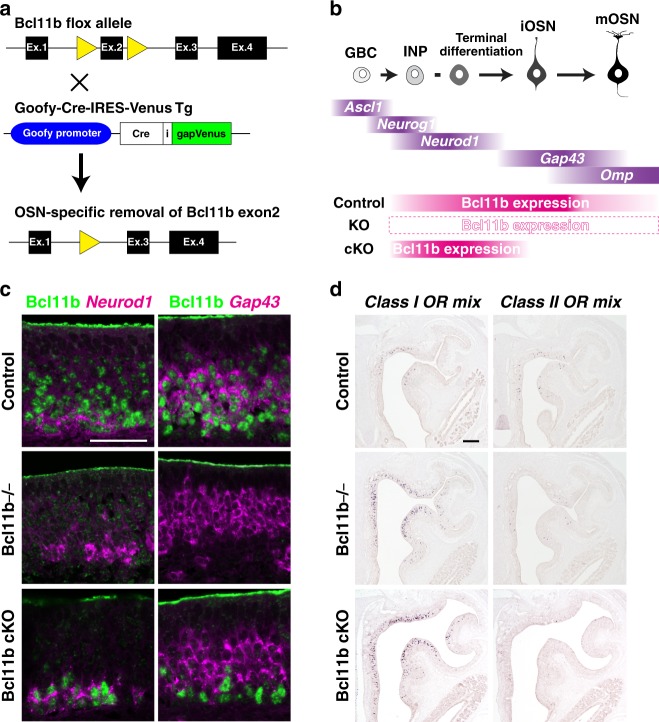
Specific depletion of Bcl11b in postmitotic OSNs switches the OR class from class II to class I. **a** Genetic strategy to generate Bcl11b cKO mice. **b** Schematic illustration of the genetic manipulation of Bcl11b expression during OSN differentiation. GBC, globose basal cell; INP, immediate neuronal precursor; iOSN, immature OSN; mOSN, mature OSN. IHC analyses of the expression of Cre recombinase during the OSN-differentiation in the MOE of Goofy-Cre-IRES-Venus Tg mice are presented in Supplementary Fig. 4. **c** Combination of IHC against Bcl11b (green) and ISH with either *Neurod1* or *Gap43* probes (magenta) on coronal sections of the MOE of control, Bcl11b−/−, and Bcl11b cKO mice. Bcl11b-immunoreactivity is detected in *Neurod1*-expressing cells but not detected in *Gap43*-expressing cells in Bcl11b cKO, indicating specific depletion of Bcl11b in postmitotic OSNs. **d** ISH with mixed RNA probes for the eight class I and class II genes in coronal sections of the control, Bcl11b−/−, and Bcl11b cKO mice. Quantification data of coexpression analyses is summarized in Supplementary Fig 4d and Supplementary Data 1. Scale bars, 50 μm in (**c**); 100 μm in (**d**)

*Bcl11b* is expressed in both the MOE and the OB (Supplementary Fig. 1a–c). Although Bcl11b−/− mice displayed no obvious abnormalities in the OB except the glomerular domain organization (Fig. 2 and Supplementary Fig. 3), the phenotypes observed in OSNs might be due to a cell-autonomous effect of the loss of the Bcl11b function, a non-cell-autonomous effect, or both. To investigate this issue, we generated conditional Bcl11b knockout (Bcl11b cKO) mice, in which Bcl11b was specifically depleted in postmitotic OSNs by expressing Cre recombinase under the control of the *Goofy* promoter (Fig. 3a–c, Supplementary Fig. 4a–c, Supplementary Data 1). We analyzed the effect of OSN-specific Bcl11b depletion on OR gene expression by ISH using mixed probes for class I and class II genes. Bcl11b cKO mice displayed the same phenotypes as Bcl11b−/− mice (Fig. 3d, Supplementary Fig. 4d and Supplementary Data 1), indicating that the phenotypes observed in Bcl11b−/− OSNs were due to a cell-autonomous effect of the loss-of-function of Bcl11b. Importantly, because the ventral MOE is occupied by class II OSNs in wild type mice, our experiments reveal that immature OSNs destined to express class II OR genes are capable of instead expressing class I OR genes. Considering that OR gene expression starts in GAP43-positive immature OSNs^26^, the OR class choice can take place at the latest at the immature stage.

### Bcl11b negatively regulates the class I OR gene expression

**Fig. 4:**
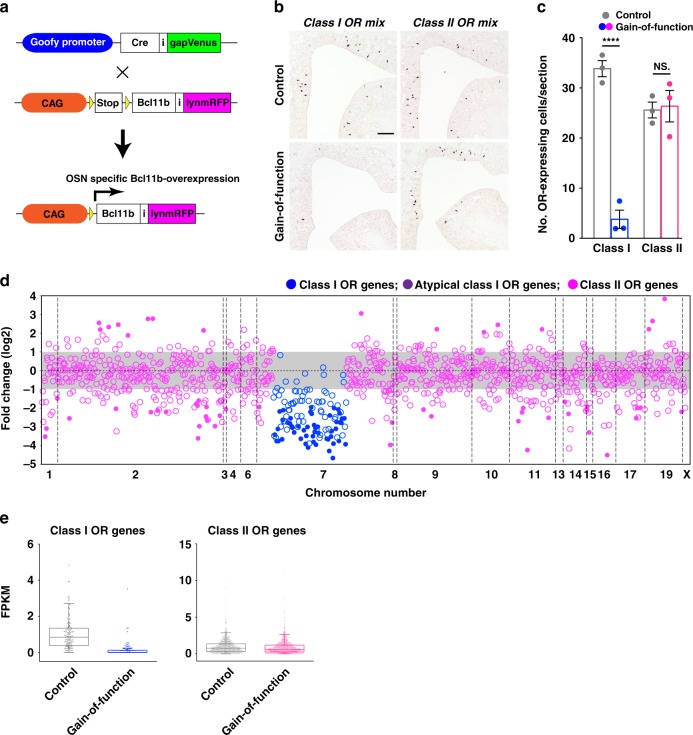
Bcl11b negatively regulates the expression of class I OR genes. **a** Genetic strategy of the gain-of-Bcl11b-function analysis in OSNs. Robust expression of Bcl11b throughout the MOE of the gain-of-function mutant mice was confirmed by IHC (Supplementary Fig. 5a). **b** ISH with mixed RNA probes for the four class I and dorsal class II genes in coronal sections of the MOE of the control and gain-of-function mutant mice. Scale bar, 100 μm. **c** Quantification of the number of OSNs expressing either class I or class II genes per section. Bars represent the mean values ± s.e.m. of the control (gray) and gain-of-function mutant mice (class I in blue, class II in magenta), respectively: Class I genes: 33.8 ± 1.60 in control and 3.78 ± 1.82 in mutant mice, *p* = 0.000243, two-tailed *t*-test, *n* = 3 independent experiments; Class II genes: 25.6 ± 1.57 in control and 26.4 ± 3.15 in mutant mice, *p* = 0.838, two-tailed *t*-test, *n* = 3 independent experiments. ****p < 0.001, NS, not significant. Quantification data and statistical details are summarized in Supplementary Data 1. **d** The log_2_-fold change values of OR gene expression analyzed by RNA-seq are arranged according to their relative positions along the chromosomes. Class I genes (blue), atypical class I genes (purple), and class II genes (magenta). Increased- and decreased OR genes (*p* < 0.05) are represented by filled circles. FPKMs in the control and gain-of-function mutant MOEs are summarized in Supplementary Data 3. **e** Merged representation of the bee-swarm and box-plots of RNA-seq FPKM values for class I (*n* = 128) and class II genes (*n* = 968) of the control (gray) and gain-of-function mutant (class I genes in blue; class II genes in magenta) mice

The loss-of-function analyses suggest that Bcl11b acts as a repressor for class I OR genes and/or as an activator for class II OR genes. To elucidate the molecular mechanisms underlying the function of Bcl11b in OR class determination, we designed a gain-of-function experiment such that Bcl11b-negative class I OSNs expressed Bcl11b. For this purpose, we generated Bcl11b-overexpressing Tg mice, in which Bcl11b was specifically expressed in both class I and class II OSNs (Fig. 4a). In Bcl11b gain-of-function mutant mice, we observed robust expression of Bcl11b in the OSN layer throughout the MOE (Supplementary Fig. 5a), confirming that Bcl11b was expressed in both class I and class II OSNs. Interestingly, the number of class I-expressing OSNs was reduced in the gain-of-function mutants, whereas that of class II was unchanged (Fig. 4b, c and Supplementary Data 1). IHC analysis showed that the class I-OSN axonal projection domain in the OB, called the DI domain (defined as a colabelled region with an antibody against NQO1 and Dolichos biflorus Agglutinin (DBA) lectin)^34^ was observed in control mice, but not in Bcl11b gain-of-function mutant animals (Supplementary Fig. 5b).

We further performed a comprehensive analysis of the effect of the Bcl11b gain-of-function mutation on the expression frequency of OR genes by comparing the mRNA abundance of each OR gene in the mutant and control mice using RNA-seq analysis (Fig. 4d and Supplementary Data 3). The RNA-seq data indicated that the mRNA levels of most class I ORs were perturbed in mutant mice, whereas those of most class II genes were unchanged (Fig. 4d, e). All of the changed class I ORs were downregulated, whereas those of class II genes were both downregulated and upregulated. These results of the quantification and RNA-seq analyses suggest that Bcl11b functions as a repressor for class I genes, and that class I and class II genes are differently affected by the Bcl11b gain-of-function mutation. However, since in the Bcl11b gain-of-function mutants the expression of Bcl11b was not downregulated but persisted into mature OSNs, the abnormal expression of Bcl11b might have altered transcription of ORs via different mechanisms.

### Bcl11b suppresses the J element

**Fig. 5:**
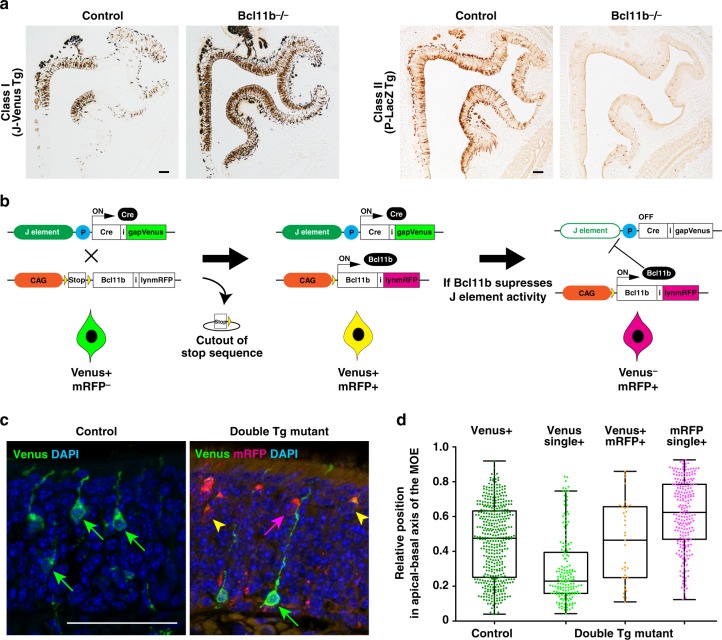
Bcl11b suppresses the enhancer activity of the J element. **a** Class I and class II enhancer activities were visualized using J-Venus and P-LacZ Tg mice in the presence or absence of Bcl11b by IHC against Venus and β-gal in coronal sections of the MOE of J-Venus and P-LacZ Tg mice, respectively. **b** Genetic strategy to analyze the functional relationship between Bcl11b and the J element. If there is no functional relationship between the induced Bcl11b and the J element, the OSN should be colored yellow by Venus (green) and mRFP (magenta). Alternatively, if the induced Bcl11b negatively regulates the J element, the expression of gapVenus will be turned off and OSNs will be colored magenta by mRFP. IHC analyses of class I OSN-specific expression of Venus in J-Cre-IRES-Venus Tg mice were shown in Supplementary Fig. 6. **c**, IHC against Venus (green) and RFP (magenta) in the MOE of J-Cre-IRES-Venus Tg mice without and with the CAGp-LSL-Bcl11b-IRES-mRFP transgene. Green and magenta arrows indicate Venus- and mRFP-single positive cells, respectively. Yellow arrowheads indicate Venus/mRFP-double positive cells. **d** Merged representation of the bee-swarm and box-plots for the relative position of OSNs along the basal-apical axis (0–1.0) of the MOE. Venus-positive cells (433 cells in control, 200 cells in double Tg mutant) and/or RFP-positive cells (251 cells in double Tg mutant) collected from three animals of each mutant strain. Quantification data are summarized in Supplementary Data 1. Scale bars, 100 μm in (**a**); 50 μm in (**c**)

Because singular OR gene expression is activated by an associated enhancer, we hypothesized that the OR class choice of OSNs could be established at the level of OR enhancer activation. To examine this hypothesis, we generated both J-Venus and P-LacZ Tg mice in a Bcl11b−/− background. In J-Venus Tg mice, a class I OSN-specific enhancer, the J element, drives gapVenus expression^11^. In P-LacZ Tg mice, the P sequence, a class II OSN-specific enhancer, drives tauLacZ expression^10^. IHC revealed that Venus-positive class I OSNs increased in number and were distributed throughout the MOE in the Bcl11b−/− background, whereas LacZ-positive class II OSNs numbers dramatically decreased (Fig. 5a), indicating that the class I enhancer was activated throughout the MOE in the absence of Bcl11b, whereas the class II enhancer was suppressed. These results suggest that Bcl11b regulates the choice between class I- and class II-enhancers to specify the OR class.

To further elucidate the molecular mechanism underlying the negative effect of Bcl11b on class I gene expression, we examined the functional relationship between Bcl11b and the J element. We generated J-Cre-IRES-Venus Tg mice for this purpose, in which the J element drives the expression of Cre and gapVenus specifically in class I OSNs (Supplementary Fig. 6 and Supplementary Data 1). We generated double Tg mice by crossing J-Cre-IRES-Venus Tg with CAGp-LSL-Bcl11b-IRES-mRFP Tg mice (Fig. 5b). In J-Cre-IRES-Venus Tg mice in the control genetic background, Venus-positive OSNs were found from the basal to the apical portion of the OSN-layer, indicating that the J element was active throughout the mature differentiation of class I OSNs. In contrast, we detected Venus-single positive OSNs primarily in the basal portion of the OSN-layer and mRFP-single positive OSNs only in the apical portion of the OSN-layer of double Tg mice, indicating that J-induced Bcl11b turned off Venus expression by suppressing the J element (Fig. 5c, d). These results reveal that Bcl11b negatively regulates the expression of class I genes by suppressing the enhancer activity of the J element.

### Biased OR class choice affects innate olfactory behaviors

Bcl11b cKO mice and Bcl11b gain-of-function mutant mice developed normally and were healthy; therefore, we were able to examine the effects of the biased populations of class I- and class II-OSNs on mouse olfactory behaviors. The expression of class I genes increased in the MOE of adult Bcl11b cKO mice, and the expression of class II genes decreased severely (Supplementary Fig. 7a, b), as observed at P0. In contrast, the expression of class I genes drastically decreased in Bcl11b gain-of-function mutant mice, while class II genes were basically unchanged (Supplementary Fig. 7c, d). Thus, we may state that the loss-of-function and gain-of-function mutant mice have class I-dominant and class II-dominant noses, respectively. To dissect the physiological function of these two nose types, we chose two odorous compounds, 2-methylbutyric acid (2MBA) and 2,5–dihydro–2,4,5–trimethylthiazoline (TMT), which elicit innate aversion in mice^14^. Interestingly, they are shown to be processed by distinct pathways; 2MBA, an odorant produced by decaying food is processed via the DI domain in the OB innervated by class I OSNs, whereas TMT, a predator odor is processed via the DII domain innervated by dorsal class II OSNs.

**Fig. 6:**
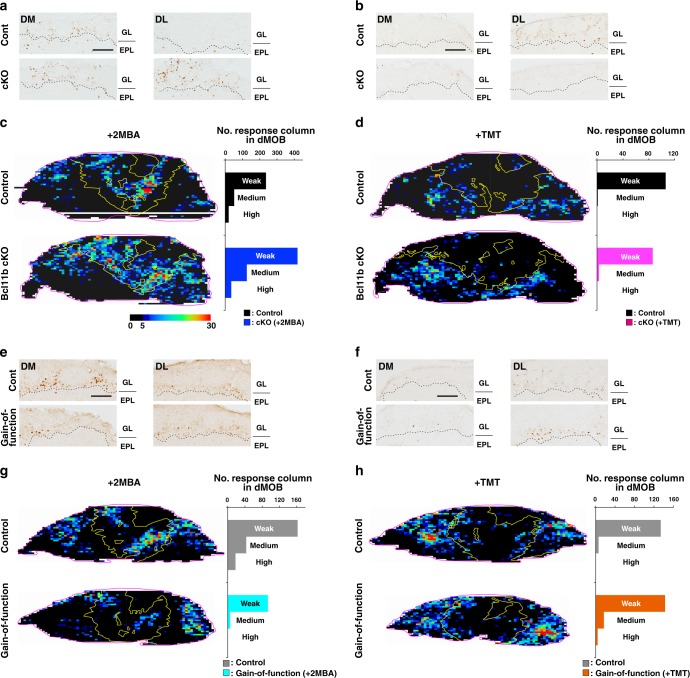
OSN-specific depletion and overexpression of Bcl11b alters physiological responses to aversive odorants. **a**–**d** Odor-responsive glomeruli in the MOB of control and Bcl11b cKO mice stimulated with 2MBA (**a**, **c**) and TMT (**b**, **d**). IHC against protein of the immediate early gene Egr1 in the dorsal-medial (DM) and dorsal-lateral (DL) regions of the MOB is shown in (**a**, **b**). The dotted lines indicate the boundary of the glomerular layer (GL) and the external plexiform layer (EPL). Unrolled odor maps for the expression of Egr1 in the GL are shown in (**c**, **d**). The magenta and yellow lines indicate the outline of maps and the boundary of Ocam-positive and negative, respectively. The color code indicates the blue to red color with correspond to the number of Egr1-positive cells in a single column. Each graph in **c**, **d** shows the histogram for the number of response column (Weak, 5~15; Medium, 15~25; High, more than 25 positive cells in a single column) of Ocam-negative dorsal MOB in Bcl11b cKO (blue for 2MBA-stimulation; magenta for TMT-stimulation) and control (Black). **e**–**h** Odor-responding glomeruli in the MOB of control and Bcl11b gain-of-function mutant mice stimulated with 2MBA (**e**, **g**) and TMT (**f**, **h**). Representation of the panels and graph in (**e**–**h**) is same as in (**a**–**d**). Bcl11b gain-of-function mutant (cyan for 2MBA-stimulation; orange for TMT-stimulation) and control (gray) are shown in (**g**) and (**h**), respectively. Reconstitution of unrolled odor maps from the immunostaining images of Egr1 and Ocam is represented in Supplementary Fig. 8. DM, dorsal-medial; DL, dorsal-lateral; GL, glomerular layer; EPL, external plexiform layer. Scale bar, 100 μm

First, to examine physiological odor responses in the MOB, we visualized the glomeruli activated following odor exposure by measuring the expression of the immediate early gene *Egr1* and reconstituting the corresponding odor maps (Supplementary Fig. 8). Odor stimulation with 2MBA activated a larger number of dorsolateral glomeruli in class I-dominant mutants relative to controls (Fig. 6a, c), but a smaller number in class II-dominant mutants (Fig. 6e, g). In contrast, the TMT-responding dorsal glomeruli decreased in class I-dominant mutants (Fig. 6b, d) but were unchanged in class II-dominant mutants (Fig. 6f, h) compared with controls.

**Fig. 7:**
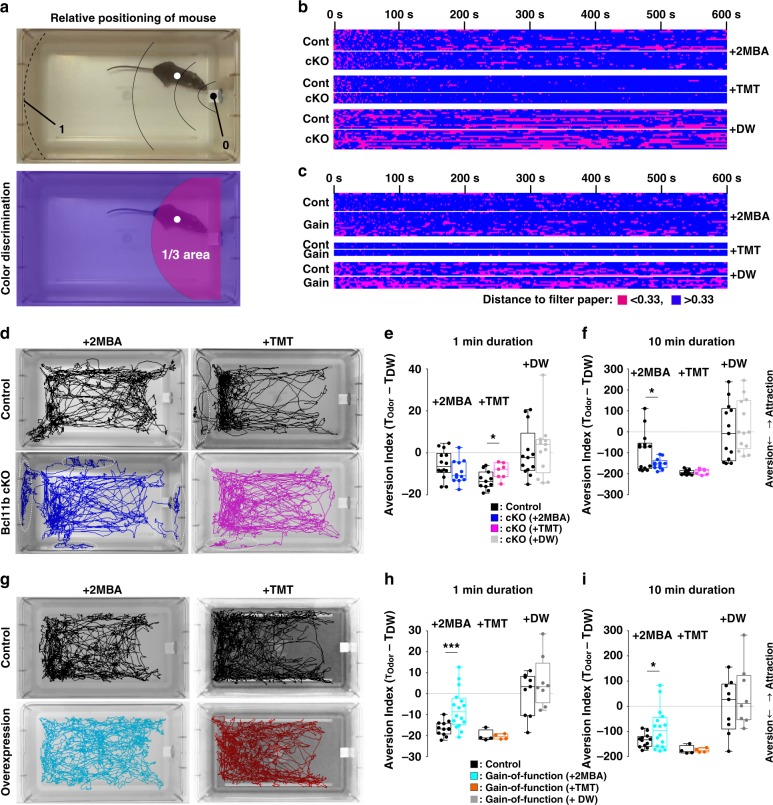
OSN-specific depletion and overexpression of Bcl11b alters behavioral responses to aversive odorants. **a** A video frame of the behavioral test, in which a male mouse is exposed to a filter paper impregnated with a particular aversive odor. Centre of a filter paper and the opposite side to a filter paper were determined to ‘0’ (black dot) and ‘1’ (dotted line), respectively. The white dot indicates center of the mouse body excluding tail. **b**, **c** Raster plots representing occupancy of each animal in two areas (magenta or blue) during the 10-min test period (x-axis) of Bcl11b cKO mice (*n* = 12 animals for 2MBA, *n* = 7 for TMT, *n* = 13 for DW) and control (*n* = 14 for 2MBA, *n* = 11 for TMT, *n* = 13 for DW) (**b**) and Bcl11b gain-of-function mutant (*n* = 16 for 2MBA, *n* = 4 for TMT, *n* = 8–9 for DW) and control (*n* = 12 for 2MBA, *n* = 4 for TMT, *n* = 9 for DW) (**c**). The two-color representation corresponds to color discrimination in (**a**). The graph of time bins is presented in Supplementary Fig. 9. **d** Representative trajectory plots of mouse positioning during the 10-min test period to 2MBA (black: control; blue: Bcl11b cKO) and TMT (black: control; magenta: Bcl11b cKO). Dotted circles indicate that mice tried to escape from the cage by climbing walls. **e**, **f** Aversion index of control (black) and Bcl11b cKO (blue: 2MBA; magenta: TMT; gray: DW) mice during the first 1 min (**e**) and 10-min (**f**) of test period. Each bar indicates merged representation of the bee-swarm and box-plots. *p < 0.05 (two-tailed *t*-test). **g** Representative trajectory plots of mouse positioning during the 10-min trials to 2 MBA (black for control; cyan for Bcl11b gain-of-function mutant) and TMT (black for control; orange for Bcl11b gain-of-function mutant). **g** Aversion index of control (black) and Bcl11b gain-of-function mutant (cyan: 2MBA; orange: TMT; gray: DW) mice during the first 1-min (**h**) and 10-min (**i**) of test period. Each bar indicates merged representation of the bee-swarm and box-plots. *p < 0.05, ***p < 0.005 (two-tailed *t*-test). All behavioral analysis data are summarized in Supplementary Data 1

Next, we tested the innate avoidance behaviors of the mutant mice (Fig. 7a). Class I-dominant mutants spent much more time in the area of the cage farthest from the odor source than control animals during a 10-min test period with 2MBA, displaying thus stronger aversion. In contrast, during the initial 1 min of the 10-min test period with TMT, class I-dominant mutants displayed a weaker aversive response to TMT than controls. However, there was no significant difference between class I-dominant mutants and controls in the aversion index during the 10-min test period, suggesting that class I-dominant mutants exhibit a delayed avoidance response to the predator’s odor (Fig. 7b, d–f and Supplementary Fig. 9a and Supplementary Data 1). In contrast to the strong aversive response of class I-dominant mutants to 2MBA, class II-dominant mutants showed a weaker aversive response. We did not observe any significant difference in aversion to TMT between class II-dominant mutants and controls (Fig. 7c, g–i and Supplementary Fig. 9b, Supplementary Data 1). These results indicate that the OR class choice of OSNs affects not only physiological odor responses but also innate olfactory behaviors.

## Discussion

**Fig. 8:**
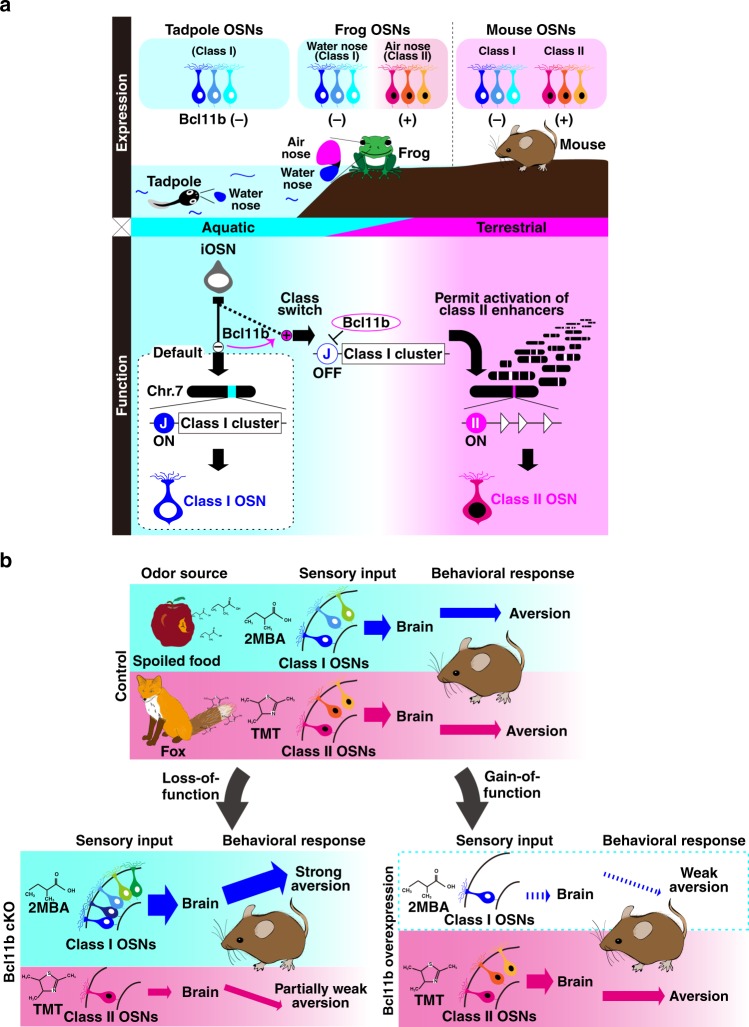
Model of the OR class specification of OSNs and terrestrial adaptation. **a** Schematic representation of the class-specific expression of *Bcl11b* in the OE of frog and mouse and the molecular mechanism of the OR class specification of OSNs. In tadpoles, the OE called “water-nose” expresses class I genes. During metamorphosis, the OE undergoes remodeling to form two distinct OE, called “water nose” and “air nose”, and Bcl11b starts expressing in the future air nose to allow the expression of class II ORs. In mice, class I and class II OSNs are Bcl11b-negative and -positive, respectively, as in adult frog. In mouse OSNs, the class I OR enhancer J-element is active in the absence of Bcl11b, whereas the presence of Bcl11b suppresses the J-element enhancer activity to permit choosing a functional class II enhancer from the class II enhancer repertoire spread through chromosomes to activate transcription of class II OR gene. **b** Schematic representation of changes in olfactory behavior caused by the biased OR class choice of OSNs. Behavioral outputs against two distinct aversive odorants, 2MBA (spoiled foods odor) and TMT (predators’ odor) depend on the populations of class I and class II OSNs. 2MBA, which is mainly detected by class I OSNs, induces stronger aversive response in Bcl11b cKO mice (class I-dominant) and less aversive in Bcl11b gain-of-function mutant mice (class II dominant). Class II-responsible odorant, TMT induces weaker aversive response when the population of class II OSNs is decreased

Our studies provide genetic evidence that OSNs throughout the MOE are capable of expressing class I genes in the absence of Bcl11b. The depletion of Bcl11b, even after terminal differentiation, i.e., after progenitor cells have divided for the final time and have become immature OSNs, can switch from one OR class to another (class II to class I), indicating that postmitotic OSNs retain notable plasticity in terms of the determination of OR class. In addition, the forced expression of Bcl11b in postmitotic OSNs reduced the class I OSN population but did not affect the class II population, indicating that Bcl11b is necessary but not sufficient to activate class II gene expression, and suggesting that unidentified factors downstream of Bcl11b are required for transcriptional activation of class II genes. Taken together, we propose that class I is a default class of OR to be expressed in OSNs and that Bcl11b is a binary switch to select OR class. In this model, OSNs choose the class I gene by default in the absence of Bcl11b, and in the presence of Bcl11b, class I expression is suppressed, thereby allowing class II gene choice (Fig. 8a).

In the class I-default model, OSNs must choose class I genes preferentially in the absence of Bcl11b. Recently, Magklara et al. demonstrated that class II loci possess the unusual molecular hallmarks of constitutive heterochromatin (H3K9me3 and H4K20me3) prior to the OR choice to silence class II OR gene expression, and proposed a model termed stochastic escape, in which a singular OR gene might be chosen by stochastic escape from heterochromatic silencing^35^. Intriguingly, the level of constitutive heterochromatin is lower in class I loci than class II, suggesting that class I genes are chosen preferentially. The class I default model and the stochastic escape model for class II genes complement each other. It is possible to construct a scenario of class determination and subsequent singular OR gene choice by combining the two models. In the mouse genome, ~1100 OR genes are scattered over 69 loci (a single cluster of class I and multiple clusters of class II), and a given OSN does not choose a single OR allele randomly from these loci, but rather there is a hierarchy of the OR class choice prior to singular OR gene expression. OSNs chooses the OR class depending on Bcl11b. In the absence of Bcl11b, OSNs choose class I as the default class. The class I choice is suppressed in the presence of Bcl11b, which in turn permits the choice of a single class II gene through stochastic escape from heterochromatic silencing.

Our previous studies have shown that deletion of the *trans* J element increases the expression frequency of class I genes from the intact J allele, indicating that singular class I gene expression is not solely established by the stochastic choice of a single class I allele but is rather orchestrated with the choice of a functional enhancer^11^. Our study demonstrated that the enhancer activity of the J element for class I genes is active throughout the MOE in the absence of Bcl11b, whereas that of the P element for class II genes is suppressed (Fig. 5a), indicating that the class choice of OSNs is determined at the OR enhancer level. Furthermore, our experiments provide experimental evidence that the presence of Bcl11b negatively regulates the expression of class I genes by suppressing the enhancer activity of the J element. Together, we identify a unique transcriptional mechanism underlying OR class choice, in which the J element can function not only as a gene choice element for class I but also as a class choice element in combination with Bcl11b. Although it is not known whether Bcl11b suppresses the J element by direct binding or the recruitment of associated proteins, our results demonstrate that the deficiency of Bcl11b does not affect the expression of transcription factors recruited to OR enhancers and promoters^36–38^, including Lhx2, Emx2, and Ebf family proteins (Supplementary Data 2). This suggests that the suppressive effect of Bcl11b on the J element is not mediated by other transcription factors that are enriched on OR enhancers/promoters.

Because the most recent common ancestor of vertebrates lived in water, one can assume that class I is a default- or proto-type of OR. Despite this assumption, no experimental evidence has been provided. In this study, we provide evidence that class I is a default type of OR expressed in OSNs. The class I default model can be applied to understand the terrestrial adaptations of olfaction. During vertebrate evolution, there was an enormous expansion in the number of class II genes in amphibian and mammalian lineages, most likely as an adaptation to terrestrial environments. OSNs acquired the ability to express class II genes at the water-to-land transition^2^. Such a drastic transition can be observed in a single animal. In frogs, the OE undergoes massive remodeling during metamorphosis from aquatic environments in larvae to semiaquatic or terrestrial environments in adults^39^. The class selection of OR genes in the frog OE is ingeniously regulated from aquatic to terrestrial olfaction. In tadpoles, the OE in the principal cavity (PC) initially expresses class I genes (stage 40, Supplementary Fig. 10a). During metamorphosis, tadpoles rearrange the PC into an adult “air-nose” to start expressing class II genes, and a newly formed “water-nose” in the middle cavity (MC) expresses class I genes (stage 52, Supplementary Fig. 10a). In adults, class I genes are exclusively expressed in the MC (water-nose), whereas class II genes are expressed in the PC (air-nose)^6,40^.

To examine our hypothesis regarding the function of Bcl11b in the acquisition of terrestrial-specific class II gene expression, we examined the expression of *Xenopus tropicalis Bcl11b* (*XtBcl11b*) in the frog OE. Interestingly, *XtBcl11b* is expressed in the frog OE, and its expression patterns dynamically change during metamorphosis, consistent with that of class II genes. *XtBcl11b* expression is initiated in the future air nose during metamorphosis, and specific expression of XtBcl11b in the air nose is obvious after metamorphosis. Moreover, double-labeling analysis revealed that XtBcl11b is coexpressed predominantly with class II genes and not class I genes (Supplementary Fig. 10b–e). Although the J element has not yet been identified in amphibian lineages, these results suggest that XtBcl11b functions as a suppressor for class I genes in frogs in the same manner as we have demonstrated in mice. Thus, it is conceivable that the aquatic larval PC expresses class I genes as a default state, and that the expression of XtBcl11b in the metamorphic and adult air nose permits the expression of class II genes for terrestrial olfaction.

In this study, we also demonstrated that the biased populations of class I and class II OSNs in Bcl11b cKO and Bcl11b gain-of-function mutant mice affect physiological and behavioral responses to the two aversive odorants 2MBA (spoiled food odor) and TMT (predators’ odor) in opposite ways (Fig. 8b). The antithetical effects against two distinct aversive odorants can be explained at the peripheral level primarily through the biased class I and class II OSN population. In addition, the changes in glomerular domain organization due to the biased populations of class I and class II OSNs may also be responsible for the abnormal olfactory behaviors. In Bcl11b cKO mice, 2MBA stimulation activates both the DI and DII domains in the OB and induces a stronger aversive response. If the neural circuits from DI and DII glomeruli to the higher order neurons in the brain are hard-wired as shown in the *Drosophila* olfactory system^41^, the stronger aversive response observed in Bcl11b cKO mice can be interpreted to be due to simultaneous activation of both the DI and DII pathways. Alternatively, if the neural circuits are soft-wired, i.e., primarily determined by the neuronal identify of OSNs, the stronger aversive response is simply due to the increased class I OSN population. The olfactory processing pathway in the higher olfactory centers of these mutants in response to the two aversive odorants currently remains unknown. Further studies examining the functional glomerular organization and olfactory processing pathway in the class I-dominant and class II-dominant mice will unveil this interesting issue.

Our present study identifies Bcl11b as a critical regulator and reveals the molecular mechanism of OR class choice. Genetic manipulations of Bcl11b generate mice with “class I-dominant” and “class II-dominant” noses, mice which display abnormal innate aversive behaviors, demonstrating that alterations of the OR class choice in peripheral OSNs change odor perception in mice. Overall, these findings reveal a unique transcriptional mechanism that serves as a binary switch for OR class choice that is crucial to both the anatomical and functional organization of the olfactory system.

## Methods

### Animals

All mice were housed under standard conditions with a 12 h light–dark cycle and access to water and food ad libitum. Information of previously described gene-targeting mouse strains and transgenic mouse strains is provided in Supplementary Data 4. Detailed information for other mouse strains generated in this study is described in the following sections. The conventional Bcl11b knockout mouse strain (Bcl11b−/−)^28^ was maintained on a BALB/c genetic background, and other strains had a mixed genetic background. Mutant and wild-type mice/embryos of either sex were used. For embryo staging, mid-day of the day of the vaginal plug was designated as embryonic day (E) 0.5. The day of birth was designated postnatal day (P) 0.

Larval and young postmetamorphic *Xenopus tropicalis* (stages 47–60) were provided by the Institute for Amphibian Biology, Hiroshima University, through the National Bio-Resource Project of MEXT, Japan. Staging was designated according to Nieuwkoop and Faber^42^. All animal studies were approved by the Institutional Animal Experiment Committee of the Tokyo Institute of Technology and were performed in accordance with institutional and governmental guidelines.

### Generation of mouse strains

The transgene of *CAGp-LSL-Bcl11b-IRES-lynmRFP* was constructed by inserting a *Bcl11b* cDNA into the Cre-dependent expression vector, consisting of the CAG promoter, *LSL* (*LoxP-STOP-LoxP*) cassette, and *IRES-lynmRFP* sequence, in which the LSL cassette is excised in the presence of Cre recombinase to induce the expression of Bcl11b and a fluorescent marker lynmRFP. Transgenic lines were generated by microinjecting the purified transgene into B6C3F1 pronuclei. Genotyping were carried out by PCR to amplify the 264-bp product of the mRFP sequence. CAGp-LSL-Bcl11b-IRES-lynmRFP Tg mice (line 9) were used. For the gain of Bcl11b function analysis, CAGp-LSL-Bcl11b-IRES-lynmRFP Tg mice (line 9) were crossed with Goofy-Cre-IRES-gapVenus Tg mice^43,44^ to obtain double Tg mutants mice. In the double Tg mutants, OSN-specific expression of Cre excises a STOP signal to induce transgene expression specifically in postmitotic OSNs. Transgene expression can be monitored by lynmRFP fluorescence.

We generated transgenic mouse lines of J-Cre-IRES-gapVenus, in which the J element drives the expression of Cre recombinase and gapVenus in class I OSNs. The transgene was constructed by inserting a 3.8-kb NcoI fragment containing the J element^11^ into the reporter vector, consisting of the *Olfr544* promoter region and the *Cre-IRES-gapVenus-pA* sequence. The *Olfr544* promoter region was amplified by PCR. Transgenic lines were generated by microinjecting the purified *J-Cre-IRES-gapVenus* transgene into B6C3F1 pronuclei. Tg mice (line 13) were used. To analyze the functional relationship between Bcl11b and the J element, a class I OR enhancer, CAGp-LSL-Bcl11b-IRES-lynmRFP Tg mice (line 9) were crossed with J-Cre-IRES-gapVenus Tg mice (line 13) to obtain double Tg mutant mice.

OSN-specific Bcl11b knockout (Bcl11b cKO) mice were generated by crossing Bcl11b^flox/flox^ mice^45^ with Goofy-Cre-IRES-gapVenus Tg mice to obtain Bcl11b^flox/flox^; Goofy-Cre-IRES-gapVenus hemizygous mice. The *goofy* promoter-driven Cre excised the Bcl11b floxed allele in postmitotic OSNs. To maintain the Bcl11b cKO line, double mutant mice of Bcl11b^flox/flox^; Goofy-Cre-IRES-gapVenus hemizygous were crossed with Bcl11b^flox/flox^. Genotyping of Bcl11b cKO was carried out by PCR to amplify the 508-bp product of the Venus sequence or the 361-bp product of Cre sequence.

### In situ hybridization

Information for all riboprobes is summarized in Supplementary Data 4. Riboprobes for *Bc111b, Ascl1, Neurog1, Neurod1, Stmn2, Gap43*, *Omp*, and *Acsm4* were prepared as described previously^22,46,47^. The OR coding sequence and others were cloned from olfactory cDNA or genomic DNA into the pGEM-T easy vector (Promega, catalog #A1360) by PCR and sequenced. All RNA probes were synthesized using *in vitro* transcription by T3, T7, or Sp6 RNA polymerase (Roche, 11031163001; 10881767001; 10810274001) with hapten-labeled UTP of digoxigenin (DIG) (Roche, 11277073910) or dinitrophenyl (DNP) (PerkinElmer, NEL555001EA) in the presence of an RNase inhibitor, RNasin (Promega, N2111).

Frog and mouse tissues were fixed in 4% paraformaldehyde (PFA) in PBS at 4 °C overnight. All postnatal mice were transcardially perfused with 4% PFA in PBS. For adult mice, head samples were soaked in 0.45 M EDTA in PBS for decalcification for at least 2 days. After cryoprotection in 15 and 30% sucrose in PBS, the tissue samples were embedded in FSC 22 Frozen Section Media (Leica Biosystems, 3801481) and sectioned using a cryostat (Microm HM505E or Leica Biosystems CM3050S). Sections were collected on MAS-coated glass slides (Mastunami, S9441).

Chromogenic single-color and fluorescent two-color in situ hybridization (ISH) were performed according to previously described methods^22,48^. Briefly, the postfixed sections were pretreated according to the following procedures, permeabilization, HCl-treatment and acetylation, and hybridized with hapten-labeled probes at 65 °C overnight. After the hybridization and wash steps, we conducted chromogenic or fluorescence detection. For the chromogenic detection of single-color ISH, the sections were incubated with 1% (v/w) DIG blocking reagent (Roche, 11096176001) and an anti-DIG-AP antibody (1/1000 dilution) at room temperature for 60 min each. After washing in TN buffer (100 mM Tris-HCl of pH 7.5; 150 mM NaCl) with 0.01% Tween 20, ISH signals were detected using alkaline phosphatase chromogens, nitro blue tetrazolium (NBT) and 5-bromo-4-chloro-3-indolyl-phosphate (BCIP) (Roche, 11681451001) in DIG3 buffer (100 mM Tris-HCl of pH 9.5; 100 mM NaCl; 100 mM MgCl_2_; 0.01% Tween20) at room temperature overnight. Chromogenic stained sections were air-dried completely and mounted with Entellan New Mounting Medium (Merck Millipore, 107961). The bright-field images of the chromogenic single-color ISH were obtained on an Olympus BX51 microscope with a DP71 digital CCD camera.

For fluorescence detection of two-color ISH, after stringent washes, the sections were treated with 0.5% (v/w) TSA blocking reagent (PerkinElmer, FP1020) in TN buffer with 0.05% Tween 20, and incubated with anti-DIG-AP (1/1000 dilution) and anti-DNP-HRP (1/1000 dilution) antibodies in blocking buffer at 4 °C overnight. The DNP-labeled target was detected using a combination of donkey Alexa Fluor 488-conjugated anti-rabbit IgG (Jackson ImmunoResearch Laboratories, 711-546-152; 1/500 dilution) and rabbit anti-DNP-KLH (1/300 dilution) antibodies after amplification using the Tyramide Signal Amplification (TSA) Plus DNP System (PerkinElmer, NEL747B001KT). Subsequently, the DIG-labeled target was detected using the HNPP/FastRed detection kit (Roche, 11758888001). Polyvinyl alcohol mounting medium with DABCO (SIGMA, 10981) was used for fluorescence detection of the two-color ISH signals. To analyze the coexpression of DIG- and DNP-labeled targets in a given cell, low-magnification images throughout the MOE were obtained on an Olympus BX51 microscope with a DP71 digital CCD camera. All potentially overlapping cells were then captured at high-magnification, and the optical stacked images were checked using a Leica SPE confocal microscope. All bright-field and fluorescence images were optimized (brightness and contrast) using Adobe Photoshop CS4 software.

### Immunohistochemistry

Immunohistochemistry (IHC) was performed according to a previously described method^22^. Sampling and sectioning of olfactory tissues were carried out as described for the ISH method, except the fixation time was 30 min. Postfixed sections were permeabilized in 0.1% TritonX100 in PBS and treated with either HistoVT one (Nacalai tesque, 06380-05) or Target Retrieval Solution (Dako, S1699) in a hot bath for antigen retrieval. Blocking reagent was selected (either 10% normal horse serum (Vector laboratories, S-2000) or 5% skim milk (Megmilk Snow Brand)). Intrinsic peroxidase and avidin/biotin were blocked using the 0.3% H_2_O_2_ and Avidin/Biotin Blocking Kit (Vector laboratories, SP-2001). After blocking, the sections were incubated with primary antibodies at 4 °C overnight. The antibodies are listed in Supplementary Data 4. For chromogenic detection, biotinylated secondary antibodies and the VECTASTAIN Elite ABC HRP Kit (Vector laboratories, PK-6100) were used to visualize immunosignals. After chromogenic detection with 0.05% 3,3′-diaminobenzidine (DAB) in the presence of 0.01% H_2_O_2_, the slides were washed, air-dried, and mounted with Entellan New Mounting Medium. For fluorescence detection, the following appropriate conjugated secondary antibodies were used: Alexa series (Thermo Fisher Scientific, Molecular Probes^TM^), Cy and Dylight series (Jackson ImmunoResearch Laboratories), and CF series (Biotium). After incubation with the secondary antibodies, the sections were washed with PBS and counterstained with 2 μg/ml of 4′,6-diamidino-2-phenylindole (DAPI) solution for 3~5 min. Fluoromount (Diagnostic BioSystems, K024) or CC/Mount (Diagnostic BioSystems, K002) mounting medium was used. The TSA Biotin System (PerkinElmer) was used for amplification of immunosignals. The Mouse on Mouse (M.O.M.) Basic Kit (Vector laboratories, BMK-2202) was used to detect accommodating mouse monoclonal primary antibodies. The immunostained images were acquired on an Olympus BX51 microscope with a DP71 digital CCD camera, a Zeiss Axio Observer Z1 with an ORCA Flash 4.0 (Hamamatsu) camera and a Leica SPE confocal microscope. All bright-field and fluorescent images were optimized (brightness and contrast) using Adobe Photoshop CS4 software.

### Combination of ISH and IHC

Combined detection of ISH and IHC was performed according to the abovementioned ISH method with some modifications. For fluorescence detection of ISH and IHC, the incubation time for proteinase K treatment was reduced to 5 min in the pretreatment steps before hybridization. After the hybridization and wash steps, the sections were blocked with 0.5% (v/w) TSA blocking reagent (PerkinElmer, FP1020) and incubated with an anti-DIG-AP antibody (for ISH signals) and either rabbit anti-Bcl11b or anti-Cre antibodies (for IHC signals) at 4 °C overnight. In the case of frog MOE, the sections were treated with HistoVT at 58 °C for 60 min before blocking with 0.5% (v/w) TSA blocking reagent, followed by incubation with an anti-DIG-AP antibody (for ISH signals) and a rat anti-Bcl11b antibody (for IHC signals) at 4 °C overnight. On the following day, after a 60-min incubation with a biotinylated goat anti-rabbit IgG antibody, immunosignals were detected with Alexa Fluor 488-conjugated streptavidin (Thermo Fisher Scientific, S11223), as shown in Supplementary Fig. 1d. For amplification of immunosignals, the TSA Biotin System (PerkinElmer, NEL700A001KT) was used after incubation with POD-conjugated streptavidin (PerkinElmer, NEL750001EA), as shown in Figs. 1b, 3c, and Supplementary Fig. 4b, 10c. ISH signals were detected using the HNPP/FastRed detection kit (Roche). Slides were mounted using polyvinyl alcohol mounting medium with DABCO antifading reagent (Sigma-Aldrich, 10981). Fluorescence images were acquired on a Leica SPE confocal microscope. All fluorescence images are optimized (brightness and contrast) using Adobe Photoshop CS4 software.

### Analysis of whole mount specimens

Visualization of β-galactosidase activity with X-gal and endogenous GFP or YFP fluorescence in whole mounts has been described previously^10,33^. For X-gal staining, whole mount tissues were fixed for 30 min on ice with 4% PFA in PBS and washed with buffer A (2 mM MgCl_2_ and 5 mM EDTA in PBS) twice for 5 min at room temperature. After incubation with buffer B (2 mM MgCl_2_, 0.01% sodium deoxycholate and 0.02% Nonidet P40 in PBS) for 30 min, β-galactosidase activity was visualized with buffer C (5 mM potassium ferricyanide, 5 mM potassium ferrocyanide and 1 mg/ml X-gal in buffer B) at 37 °C overnight. Images of X-gal staining were acquired on an Olympus SZX10 stereomicroscope with a DP71 digital CCD camera. Fluorescence wholemount images were collected as z-stacks and projected into a single image using a Leica SPE confocal microscope.

### Microarray analysis

DNA microarray analysis was performed as described previously^22^. Briefly, MOEs were dissected from Bcl11b−/− and wild type mice at P0 and stored in RNAlater Stabilization solution (Thermo Fisher Scientific, Ambion^TM^ AM7020) at −20 °C. Biotinylated cRNA was synthesized from 10 ng of total RNA using the Two-Cycle Target Labeling and Control Reagents Kit (Affymetrix, 900494), and then fragmented. The fragmented cRNA was hybridized to the DNA microarrays (Affymetrix, Mouse Genome 430 2.0 Array, 900495). The microarray analyses were performed with RNA samples from six Bcl11b−/− and five wild type mice to ensure reproducibility. The microarray data were normalized linearly to the GAPDH (Probe ID: 1418625_s_at) signal for each preparation using GeneChip operating software (Affymetrix). The statistical significance of the differences of mRNA level between Bcl11b−/− and wild type was analyzed using GeneSpring version 7.3 (Agilent Technologies). For clustering analysis, the normalized signal intensities (SI) for the probe sets for OR genes and mature OSN-specific genes^25^ were extracted and imported into MeV software, Version TM4^49^. The imported SI value was renormalized among the gene row. A heat-map representation was obtained by hierarchical clustering and k-means clustering.

### RNA-Sequencing

MOEs from the Goofy-Cre-IRES-gapVenus Tg and Goofy-Cre-IRES-gapVenus; CAGp-LSL-Bcl11b-IRES-lynmRFP double Tg mice at P0 were frozen in liquid N_2_ and stored at −80 °C. The total RNA of each preparation was extracted using the RNeasy Mini Kit (Qiagen, 74104). From the total RNA, a cDNA library was prepared for each sample in a paired-end and strand-specific manner. The libraries were sequenced using an Illumina MiSeq sequencer (read-length, 75 bp; total-length, 3.6 Gbp for both samples), and all subsequent procedures were performed in silico. The adaptor sequences and low-quality regions in the raw reads were trimmed using Platanus_trim (version, 1.0.7; http://platanus.bio.titech.ac.jp/)^50^. To map the trimmed reads against the mouse reference genome (build, GRCm38.p3), TopHat2 (version, 2.0.13)^51^ was applied with the following options: the mouse genome annotation was input as a guide (-G annotation_file.gtf), and the maximum intron length was set to 100,000 (-I 100000). FPKM (fragments per kilobase of exon per million reads mapped) for all mouse genes were calculated based on the mapping results for the control and mutant samples as an indicator of expression, using Cuffdiff in the Cufflinks package (version, 2.2.1)^52^ with the following options: minimum number of alignments in a locus set to 5 (-c 5); multiread correction applied (-u). Finally, to each FPKM value of the OR genes was added pseudocount +0.1, and fold-changes were calculated by dividing the FPKM of the mutant by those of the control.

### Odor stimulation and Egr1 immunostaining

The odorants used in this study were purchased from Tokyo Chemical Industry (DL-2-methylbutyric acid, M0181, CAS#116-53-0) and from Contech (2,5–dihydro–2,4,5–trimethyl-thiazoline, 3000000368, LOT#13267). Bcl11b cKO, Bcl11b gain-of-function mutant and control male mice, aged 6–12 weeks, were used in this experiment. The mice were housed individually for at least 1 week. On the day of odor stimulation, the mice were placed in a clean cage (200 × 318 × 145 mm) with 50 g of clean bedding inside a fume hood and habituated for at least 2 h. A mouse was transferred to a stimulus cage with 50 g of clean bedding 1 h before the odor stimulation. For odor stimulation, 20 μl of undiluted 2MBA or 5 μl of undiluted TMT was applied to a 2 cm × 2 cm filter paper. Then, a piece of filter paper spotted with odorant was placed at the corner of cage, and the mice were exposed for 30 min. After 1 h, the mice were sacrificed and immediately perfused with 4% PFA in PBS. Heads were fixed in 4% PFA in PBS on ice for 30 min. After decalcification and cryoprotection, the tissues were embedded in FSC 22 Frozen Section Media. Consecutive 20-μm-thick sections were collected at 60 μm intervals throughout the OB and labeled with Egr1 or OCAM to compare the position of OCAM-positive glomeruli with Egr1 expression. Immunostaining for Egr1 was carried out according to a standard chromogenic IHC procedure without an antigen retrieval step.

### Reconstruction of unrolled odor maps

Unrolled odor maps were reconstructed as previously described^14,53^. For unrolled odor maps, we used the most typical one from the immunostained section sets in each mutant and control. In all Egr1- and OCAM-labeled sections, we defined as reference points the dorsal-medial and ventral edge in the granule cell layers and traced a line connecting the center of the glomerular layer (GL). The line was flattened by an opening at the ventral edge point as a sheet image of the GL using the straighten function in ImageJ/Fiji (NIH) software. Each GL sheet was aligned from anterior (top) to posterior (bottom) using the dorsal-medial edge as a reference line to create an overall unrolled map of Egr1- and OCAM-immunosignals. Outline (pink line in Supplementary Fig. 8a) and OCAM-positive/-negative boundary (yellow line in Supplementary Fig. 8b) were traced along the center in each sheet and the edge of consecutive OCAM-positive glomeruli, respectively. We omitted the punctate OCAM-positive glomeruli (blue circle in Supplementary Fig. 8b). Unrolled sheet images for Egr1 were converted with the appropriate threshold to binary images (8 bit B/W). After manual and automatic removal of dust and nonspecific signal (Despeckle and Watershed function) from the binary image, the position and number of Egr1-positive cells were automatically analyzed as size-filtered particles using the “Analyze Particle” function in ImageJ/Fiji. The number of Egr1-positive cells was reflected in the unrolled heatmap by the rainbow color scale every 100 μm division (Supplementary Fig. 8c). Pixels with an Egr1 signal spacer of less than 5 positive cells and denser than 30 positive cells per division were indicated in black and red, respectively. Outline and OCAM-positive/-negative boundary lines were superimposed onto the unrolled heatmap. In Fig. 6, we counted the number of columns (100 μm division) with weak (5–15 positive cells in one column), medium (15–25 positive cells in one column) and high (more than 25 positive cells in one column) expression in the dorsal MOB (OCAM-negative region including OCAM-positive/-negative boundary) of each unrolled odor map, and represented the results as a histogram.

### Behavioral test

Analysis of mouse aversive behavior to an odor source was performed according to previously described methods with some modifications^14,54^. Male Bcl11b cKO, Bcl11b gain-of-function mutant and control mice were housed in the same cage containing 2–6 littermates after weaning. Before the experiment, adult male mice, aged 10–15 weeks, were housed individually in a cage (200 × 318 × 145 mm) for at least 1 week. To record the behavior of each mouse from the beginning, a piece of filter paper (2 × 2 cm) spotted with odorants or distilled water was anchored to an air-vent joint on the wall of the experimental cage, which was surrounded by white plastic boards with a height of 31 cm to prevent escape. Direct physical contact with the filter paper was allowed. Each filter paper was spotted with each of the following solution: 20 μl of 2MBA, 5 μl of TMT, and 20 μl of distilled water. Mice were naive to these stimuli and tested only once to a given odor compound.

Our experimental procedure consisted of the following three steps: handling habituation for 2 days, training habituation for 2 days, and experiments for 1–3 days. Each step was performed once from 2–6 p.m. in the diurnal phase per day. At the handling habituation step, mice were habituated to the experimental condition for 10 min. The training habituation step was identical to the experimental step except for the application of distilled water as substitute for the odorants. The experiments were performed inside a fume hood. Before a trial of the experimental step, mice were placed in a clean cage with bedding and habituated to the experimental environment for 2 h. In the experimental step, each mouse was introduced to the experimental cage and allowed to habituate for 10 min. Then, the mice were transferred to the water trial cage, in which a filter paper with 20 μl of distilled water was placed. After the water trial for 10 min, the mice were transferred to the test cage for the odor trial and exposed to the odor for 10 min. Mouse behaviors were recorded during the experimental step with a digital video camera (Sony, HDR-CX420). Movies were recorded at a speed of 25 frame/s (fps) with a frame size of 320 × 180 pixels, and they were imported into ImageJ/Fiji as sequential multi tiff-images. All images were converted by autothresholding to binary images (8 bit B/W), to which were added a blur filter (3 pixels) to remove the tail. After regeneration of the binary image, the center coordinate of size-filtered particles of the mouse body was automatically analyzed using the Mtrack2 plugin in ImageJ. The distance between the center of the mouse body and the set point of a filter paper was calculated per frame. An aversion index was calculated as the subtraction between the time (T_Odor_) a given mouse spent in 1/3 area of the odor source when exposed to odorants and the average time spent (T_DW_) for all control mice in this area when water was present. In the aversion index representation, negative values indicated aversion, and positive values indicated attraction. Heat map plots were plotted based on the position of the mouse per frame during the 10-min test period.

### Statistical and reproducibility

Statistical analyses and graph representations were performed in Microsoft Excel, R and GraphPad Prism. No randomization method was used, and no statistical methods were used to predetermine the sample size. The sample sizes in this study were generally similar to those employed in the field. The variance was similar between groups that were statistically compared. To quantify the number of OR-expressing cells, every tenth coronal section (10 μm thickness) throughout the MOE was collected for each staining experiment, and the number of positive cells was counted in all sections (for Figs. 1f, g, 4c, and Supplementary Fig. 2g) or five sections around the largest MOE shape (for Supplementary Figs. 4c, d). To quantify the number of OMP-positive OSNs per unit, every tenth coronal section (10 μm thickness) throughout the MOE was collected for ISH with the *OMP* probe, and the number of positive cells was counted in the dorsomedial and ventromedial region (500 μm length) of the MOE in three sections around the largest MOE shape. To quantify the proportion of Bcl11b-positive expression (Fig. 1c) and coexpression with OR genes (Supplementary Fig. 4b), three - nine sections (10 μm thickness) of frog and mouse MOE were collected, and the number of positive cells was counted in all stained sections. All quantitative data (including the exact value, number of animals, p-value and the statistical test used) are summarized in Supplementary Data 1. For quantification of the dorsal-ventral distribution of OR-expressing OSNs (Supplementary Fig. 2h), ISH- or IHC-signals were plotted on unrolled maps of the septum along the dorsal-ventral axis, from the most dorsal point to the ventral edge of the MOE. The maps were normalized by length and divided into 10 equal segments. The number of positive cells was counted in each segment. In all statistical tests, *p*-values are indicated as * <0.05; ** <0.01; *** <0.005; **** <0.001.

### Reporting summary

Further information on research design is available in the Nature Research Reporting Summary linked to this article.

## Supplementary information

Supplementary Information

Description of Additional Supplementary Files

Supplementary Data 1

Supplementary Data 2

Supplementary Data 3

Supplementary Data 4

Reporting Summary

## Data Availability

The raw microarray data and RNA-seq data have been deposited in the Gene Expression Omnibus under ID codes GSE90798 and GSE118169. We present a summary of the following: quantitation and statistical analyses (Supplementary Data 1), microarray data (Supplementary Data 2), RPKM values of each OR gene in control and overexpression mice (Supplementary Data 3), critical reagents used in this study such as antibodies, riboprobes, PCR primers, mouse lines, and software (Supplementary Data 4). The data that support the findings of this study are available from the corresponding author upon reasonable request.
