# Author Correction: Bcl11b controls odorant receptor class choice in mice

**DOI:** 10.1038/s42003-019-0592-2

**Published:** 2019-09-12

**Authors:** Takayuki Enomoto, Hidefumi Nishida, Tetsuo Iwata, Akito Fujita, Kanako Nakayama, Takahiro Kashiwagi, Yasue Hatanaka, Hiro Kondo, Rei Kajitani, Takehiko Itoh, Makoto Ohmoto, Ichiro Matsumoto, Junji Hirota

**Affiliations:** 1 Center for Biological Resources and Informatics, Tokyo Institute of Technology, Yokohama, 226-8501 Japan; 2 Department of Life Science and Technology, Graduate School of Life Science and Technology, Tokyo Institute of Technology, Yokohama, 226-8501 Japan; 3 Monell Chemical Senses Center, Philadelphia, PA 19104 USA

**Keywords:** Olfactory receptors, Molecular neuroscience, Gene regulation, Cell fate and cell lineage

Correction to: *Communications Biology* 10.1038/s42003-019-0536-x, published online 07 August 2019.

In the version of this article initially published, an incorrect Gene Expression Omnibus accession code for the raw microarray data (GSE90700) was provided. The correct accession code is GSE90798. The error has been corrected in the data availability section of the article in the HTML and PDF versions.

