# Supplementary material for: Bcl11b controls odorant receptor class choice in mice
Source: Commun Biol. 2019 Aug 7;2:296. doi: 10.1038/s42003-019-0536-x (PMC6685970; doi:10.1038/s42003-019-0536-x)
Supplement: Supplementary file 1 — Supplementary Information [file 42003_2019_536_MOESM1_ESM.pdf]

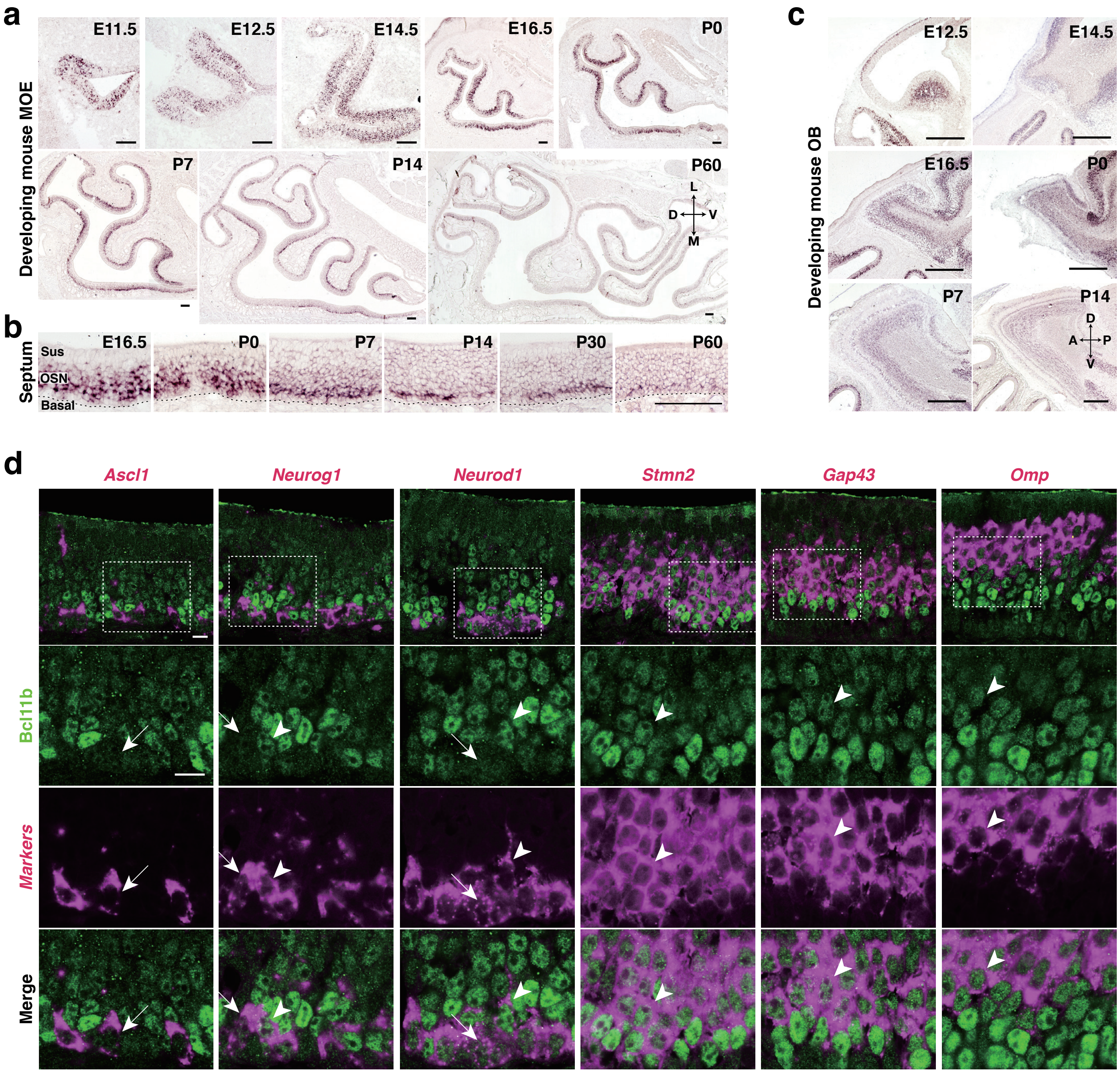

Supplementary Figure 1. The expression profile of Bcl11b in the mouse olfactory system.

(**a**, **b**) ISH with an RNA probe for Bcl11b in coronal sections of the developing mouse MOE (E11.5 to P60). D, dorsal; V, ventral; M, medial; L, lateral. The higher-magnification images of the ventromedial MOE (E16.5 to P60) are shown in b. The broken lines demarcate the olfactory epithelium and the basal lamina. SUS, sustentacular layer, OSN, olfactory sensory neuron layer. (**b**) ISH with an RNA probe for *Bcl11b* in coronal sections of the developing mouse OB (E12.5 to P14). D, dorsal; V, ventral; A, anterior; P, posterior. (**c**) Bcl11b expressing cells were characterized by combined analysis of IHC (Bcl11b, green) and ISH (magenta) with specific RNA probes for *Ascl1* (neuronal progenitors), *Neurog1* (neuronal precursors), *Neurod1* (differentiating cells and early post-mitotic neurons), *Stmn2, Gap43* (immature neurons) and *Omp* (mature neurons) in the ventral MOE at P0. Each dotted box area is magnified. Arrows and arrowheads indicate Bcl11b-negative and -positive, respectively. Scale bars, 100 µm in **a**; 500 µm in **b**; 10 µm in **c**.

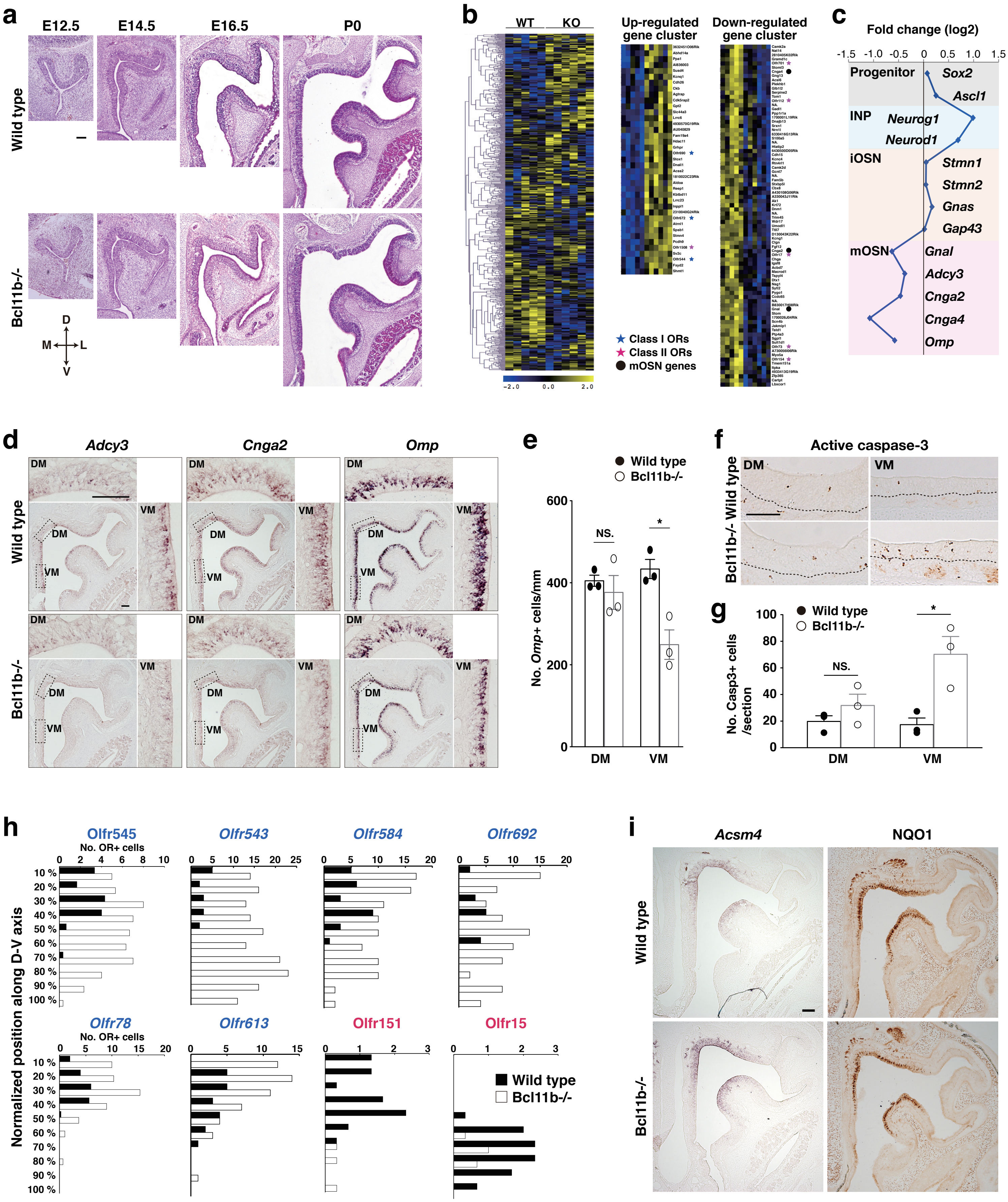

a
E12.5
E14.5
E16.5
P0
Wild type
Bcl11b-/-
D
M
L
V
b
WT
KO
Up-regulated gene cluster
Down-regulated gene cluster
Class I ORs
Class II ORs
mOSN genes
-2.0
0.0
2.0
c
Fold change (log2)
-1.5 -1.0 -0.5 0 0.5 1.0 1.5
Progenitor
Sox2
Ascl1
INP
Neurog1
Neurod1
iOSN
Stmn1
Stmn2
Gnas
Gap43
mOSN
Gnal
Adcy3
Cnga2
Cnga4
Omp
d
Adcy3
Cnga2
Omp
DM
VM
Wild type
Bcl11b-/-
e
Wild type
Bcl11b-/-
No. Omp+ cells/mm
600
400
200
0
NS.
*
DM
VM
f
Active caspase-3
DM
VM
Wild type
Bcl11b-/-
g
Wild type
Bcl11b-/-
No. Casp3+ cells /section
100
80
60
40
20
0
NS.
*
DM
VM
h
Olfr545
Olfr543
Olfr584
Olfr692
No. OR+ cells
Olfr78
Olfr613
Olfr151
Olfr15
Normalized position along D-V axis
10 %
20 %
30 %
40 %
50 %
60 %
70 %
80 %
90 %
100 %
Wild type
Bcl11b-/-
i
Acsm4
NQO1
Wild type
Bcl11b-/-

Supplementary Figure 2. Impact of Bcl11b-deficiency on the molecular and cellular organization in the MOE.

(**a**) Hematoxylin-eosin (H.E.) staining in coronal sections of the wild type and the Bcl11b-/- MOE at E12.5, E14.5, E16.5, and P0. D, dorsal; V, ventral; M, medial; L, lateral. (**b**) Hierarchical clustering of genes expressed in OSNs of the wild type (WT) and Bcl11b-/- (KO) MOE by microarray analysis. Each column refers to independent MOE samples from five wild type and six Bcl11b-/- mice. Color scale indicates the log10 value of the signal intensity normalized to the internal control, *GAPDH*. Class I OR and class II OR genes are labeled by blue and magenta star-symbols, respectively. Mature OSN (mOSN) marker genes are indicated by circle. (**c**) The log2-fold changes for neuronal differentiation marker genes in the Bcl11b-/- MOE by microarray analysis. (**d**) ISH with the RNA probes for three mOSN marker genes: *Omp*, *Cnga2* and *Adcy3* in the MOE of wild type and Bcl11b-/- mice at P0. The dorsomedial (DM) and ventrolateral (VM) regions of the MOE in each low magnification image are shown. (**e**) Quantification of the number of cells expressing *Omp* in the dorsomedial (DM) and ventromedial (VM) MOE of wild type (black circles) and Bcl11b-/- (open circles) mice. Note that the number of *OMP*-positive cells was reduced in the ventral MOE of Bcl11b-/- mice, but not in the dorsal MOE (the dorsal MOE: 405 ± 13.6 in wild type and 376 ± 41.3 in Bcl11b-/- mice, $p = 0.5482$, two-tailed *t*-test, $n = 3$; the ventral MOE: 434 ± 23.4 in wild type and 249 ± 35.9 in Bcl11b-/- mice, $p = 0.0126$, two-tailed *t*-test, $n = 3$). *$p < 0.05$ (*t*-test); NS, not significant. (**f**) IHC for active caspase-3 in the dorsomedial and ventromedial MOE of wild type and Bcl11b-/- mice at P0. (**g**) Quantification of the number of active caspase-3-positive cells in the dorsomedial (DM) and ventromedial (VM) MOE of wild type (black circles) and Bcl11b-/- (open circles) mice. The number of active caspase-3 positive cells was increased in the ventromedial region of the Bcl11b-/- MOE but not in the dorsomedial region (the dorsal MOE: 19.8 ± 4.24 in wild type and 31.8 ± 8.47 in Bcl11b-/- mice, $p = 0.2740$, two-tailed *t*-test, $n = 3$; the ventral MOE: 17.3 ± 5.03 in wild type and 70.2 ± 13.4 in Bcl11b-/- mice, $p = 0.0209$, two-tailed *t*-test, $n = 3$). *$p < 0.05$ (*t*-test); NS, not significant. (**h**) The distributions of OSNs expressing class I genes (blue) and class II genes (magenta) were quantified along the dorsal-ventral axis of the septum of control (black bar) and Bcl11b-/- (white bar) mice ($n = 3$ mice each genotype). The distributions are represented in the number of positive cells in each 10 equally divided segments of the dorsal-ventral axis. (**i**) ISH and IHC for the dorsal MOE markers, *Acsm4* and NQO1, respectively in the MOE of wild type and Bcl11b-/- mice at P0. Scale bars, 100 μm.

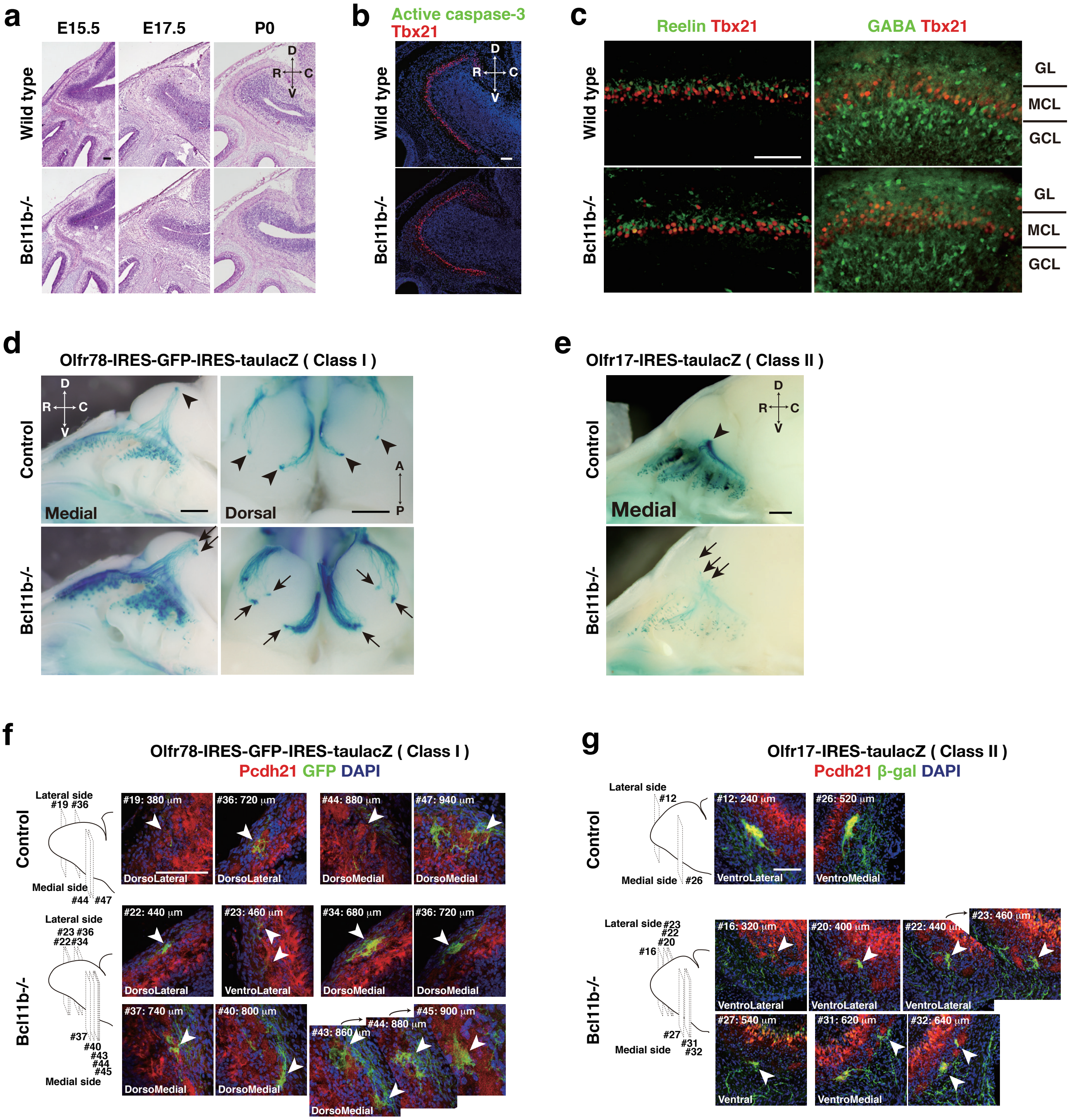

Supplementary Figure 3. Impact of Bcl11b-deficiency on the axonal projections of OSNs and the cellular and layer organization in the MOB.
(**a**) H.E. staining in sagittal sections of the OB of wild type and Bcl11b-/- mice at E15.5, E17.5, and P0. D, dorsal; V, ventral; R, rostral; C, caudal. (**b**) IHC against active caspase-3 (green) and Tbx21 (red) in the sagittal OB of wild type and Bcl11b-/- mice. (**c**) IHC against Reelin (green) or GABA (green) and Tbx21 (red) in the sagittal OB of wild type and Bcl11b-/- mice. GL, glomerular layer; MCL, mitral cell layer; GCL, granule cell layer. (**d**) Axonal projections of OSNs expressing Olfr78 to the OB. Medial view) Axonal projections of OSNs expressing Olfr78 to the OB. Medial view of the right half of the head and dorsal view of the OBs of X-gal stained wholemount preparations from Olfr78-IRES-GFP-IRES-taulacZ mice with control and Bcl11b-/- backgrounds. Arrowheads indicate the glomeruli in control. Arrows indicate the multiple glomeruli in Bcl11b-/-. (**e**) Axonal projections of OSNs expressing Olfr17 to the OB. Medial view of the right half of the head of X-gal stained wholemount preparations from Olfr17-IRES-taulacZ mice with control and Bcl11b-/- backgrounds. Arrowheads indicate the glomeruli in control. Arrows indicate the multiple glomeruli in Bcl11b-/-. (**f**) The axonal termini of Olfr78-positive OSNs in the OB of Olfr78-IRES-GFP-IRES-taulacZ mice with control and Bcl11b-/- backgrounds. The consecutive coronal sections throughout the OB were immunostained using an anti-GFP (green) and an anti-Pcdh21 (red) antibodies, and were counterstained with DAPI (blue). The section numbers of Olfr78-positive axonal termini observed are shown in the schematic diagram of OB and in each image. (**g**) The axonal termini of Olfr17-positive OSNs in the OB in Olfr17-IRES-taulacZ mice with control and Bcl11b-/- backgrounds. The consecutive coronal sections throughout the OB were immunostained using an anti-β-gal antibody (green) and an anti-Pcdh21 antibody (red), and were counterstained with DAPI (blue). The section number of Olfr17-positive axonal termini observed are shown in the schematic diagram of OB and in each image. Scale bars, 100 µm in **a**-**c**, **f** and **g**; 500 µm in **d** and **e**.

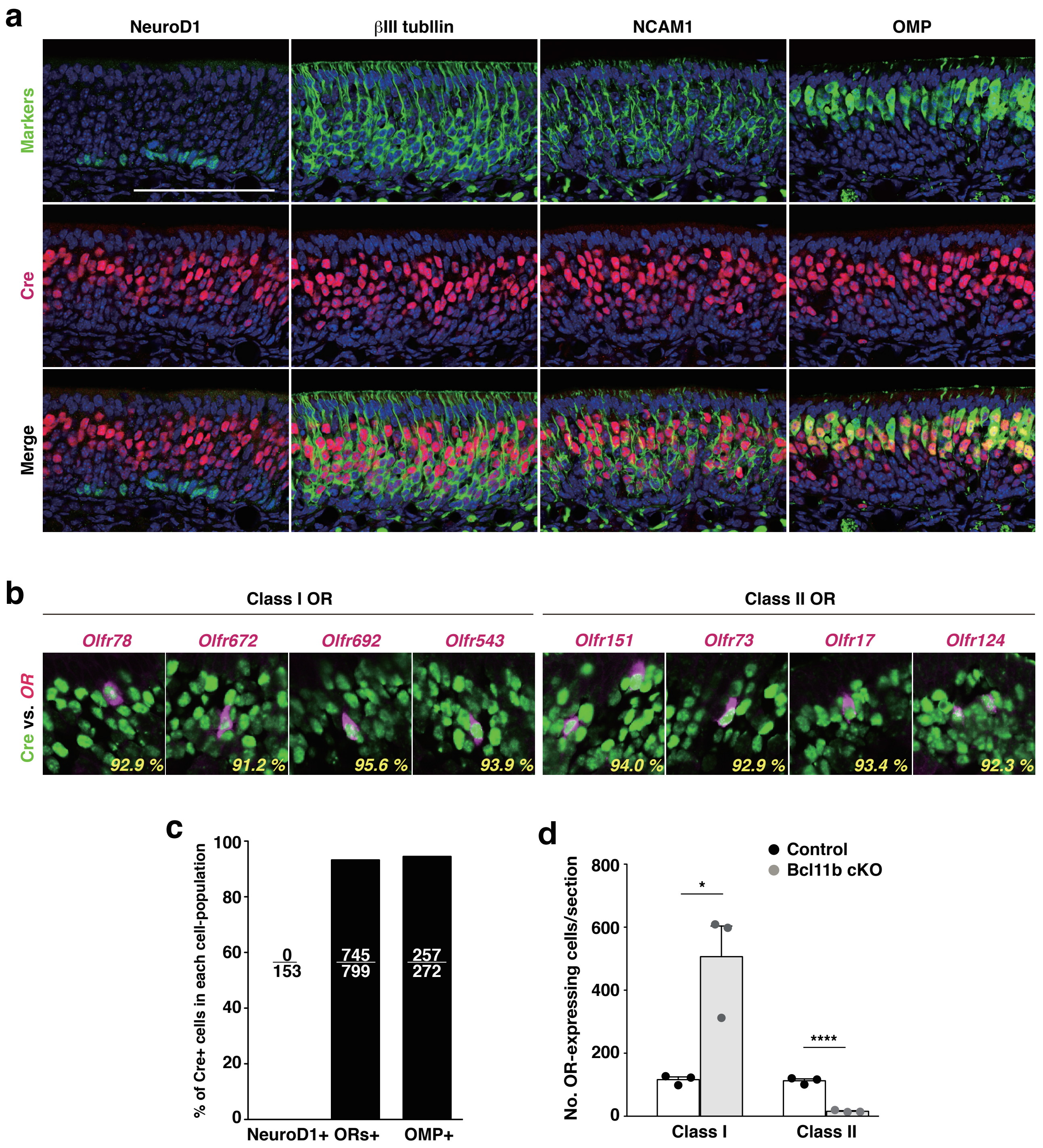

Supplementary Figure 4. The OSN-specific expression of Cre recombinase in Goofy-Cre Tg mice.
(**a**) Immunohistochemical characterization of Cre-positive cells using antibodies against neuronal differentiation markers. The coronal sections of the MOE of Goofyp-Cre-IRES-gapVenus Tg mice at P0 were immunostained with antibodies against Cre (red) and neuronal markers (green): NeuroD1, βIII-tubulin, NCAM1 and OMP, and were counterstained with DAPI. (**b**) Combination of IHC for Cre (green) and ISH for OR genes (red): Olfr78, Olfr672, Olfr692 and Olfr543 of class I genes; Olfr151, Olfr73, Olfr17 and Olfr124 of class II genes. The percentage of Cre-positive cells that co-labeled with class I or class II genes are shown in each image {92.9 % for Olfr78 (131 co-labeled cells / total 141 cells counted), 91.2 % for Olfr672 (103 co-labeled cells / total 113 cells counted), 95.6 % for Olfr692 (65 co-labeled cells / total 68 cells counted) and 93.9 % for Olfr543 (77 co-labeled cells / total 82 cells counted)} or class II {94.0 % for Olfr151 (94 co-labeled cells / total 100 cells counted), 92.9 % for Olfr73 (77 co-labeled cells / total 82 cells counted), 93.4 % for Olfr17 (114 co-labeled cells / total 122 cells counted), 92.3 % for Olfr124 (84 co-labeled cells / total 91 cells counted)}. (**c**) Bar graph shows the percentage of Cre-positive cells that were co-labeled with NeuroD1 (0 %, 0 co-labeled cells / total 153 cells counted), ORs (93.2 %, 745 co-labeled cells / total 799 cells counted), and OMP (94.5 %, 257 co-labeled cells / total 272 cells counted). The numbers of positive cells counted are as indicated. (**d**) Quantification of the number of OSNs expressing either class I or class II genes per section (control: black circles, Bcl11bcKO: grey circles). Bars represent the mean values ± s.e.m. (Class I: 116 ± 8.83 in control and 506 ± 97.2 in Bcl11b cKO mice, $p = 0.016$; class II: 113 ± 6.00 in control and 15.7 ± 1.95 in Bcl11b cKO mice, $p = 0.00011$, two-tailed t-test, $n = 3$). *$p < 0.05$; ****$p < 0.001$. Scale bar, 100 μm.

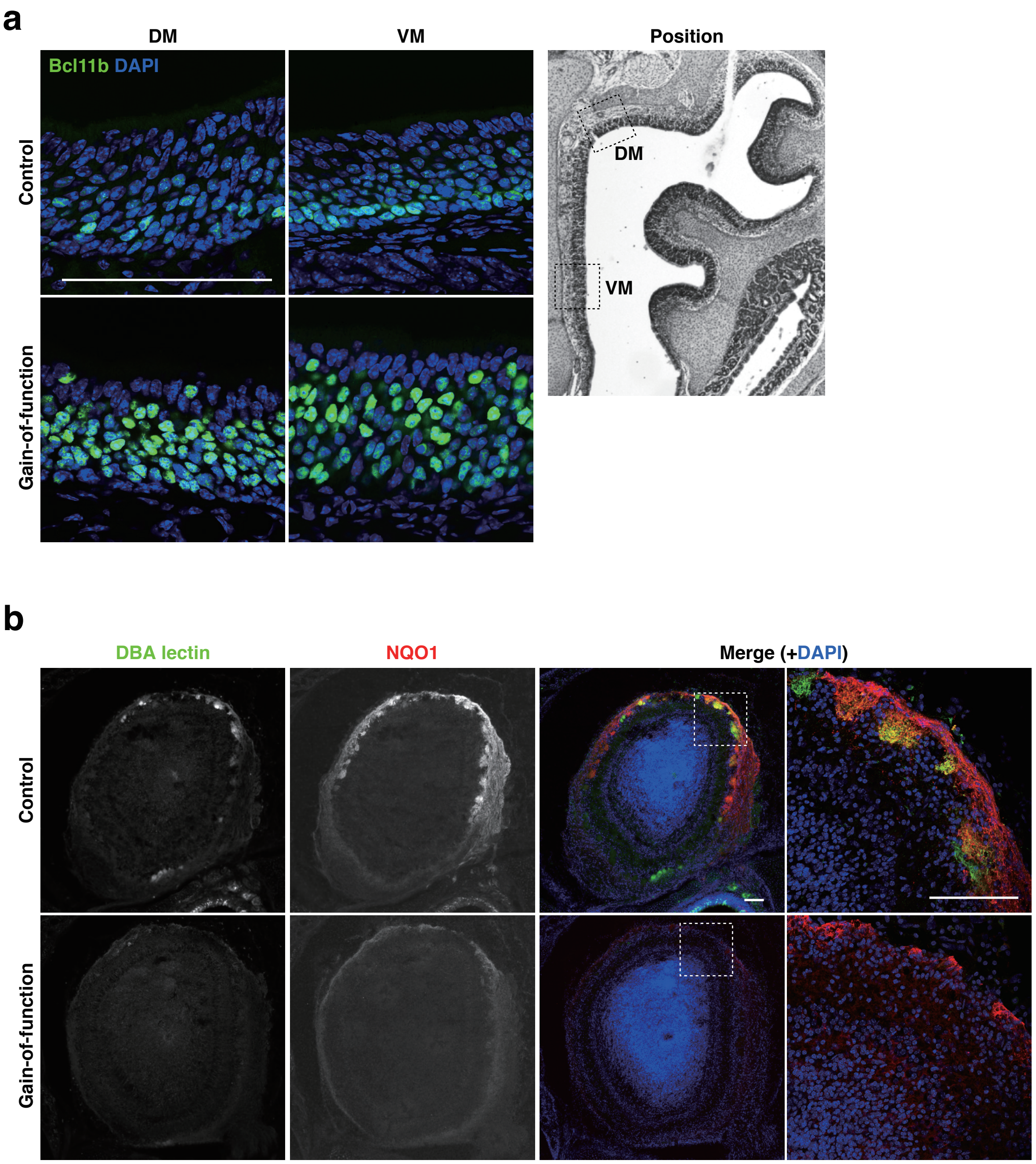

Supplementary Figure 5. Characterization of Bcl11b gain-of-function mutant mice. (**a**) Bcl11b immunofluorescence (green) in the dorsomedial and ventromedial MOE (septum) of control and Bcl11b gain-of-function mutant mice at P0. Right panel shows a low magnification image. Each dotted box area is magnified (**b**) Glomerular organization in the dorsal class I (DI) domain of the MOB. The coronal sections of the OBs of control and Bcl11b gain-of-function mutant mice were immunostained using an anti-NQO1 antibody (magenta), and were stained with DBA-lection (green). Nuclei were counterstained with DAPI. Each dotted box area is magnified. Double labeled class I glomeruli observed in the control OB are not observed in the mutant OB. Scale bars,100 μm.

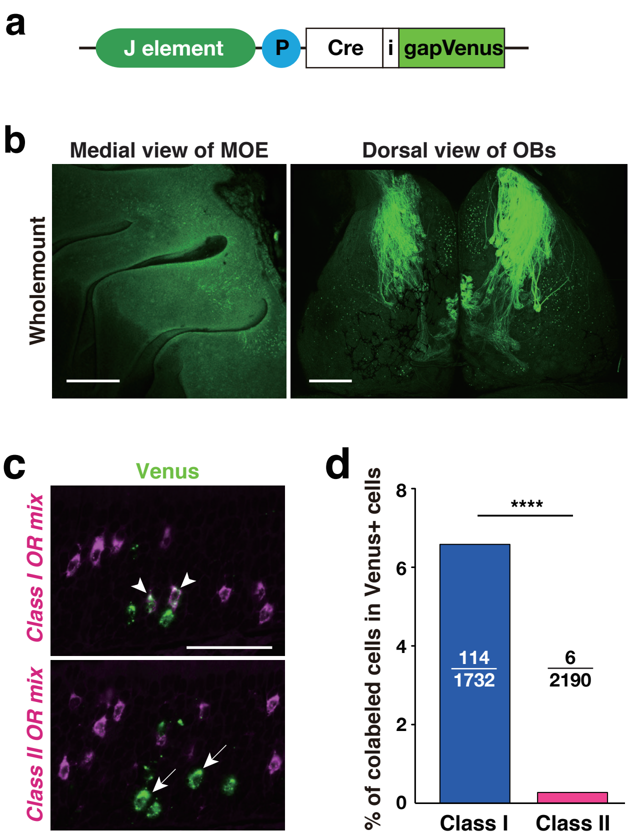

Supplementary Figure 6. Characterization of J-element active OSNs in J-Cre-gapVenus Tg mice.

(**a**) Transgene of J-Cre-IRES-Venus. (**b**) Confocal projection images of endogenous Venus fluorescence in medial view of the MOE and dorsal view of the OB of J-Cre-IRES-Venus Tg mice. (**c**) Two-color ISH with RNA probes for *Venus* (green) and mixed ORs (magenta) of five class I or five dorsal class II genes in coronal sections of the MOE of J-Cre-IRES-Venus Tg mice. Arrowheads indicate OSNs co-expressing the transgene and class I genes. Arrows indicate transgene-positive OSNs which are not co-labeled with class II probes. Scale bar 50 µm. (**d**) Bar graph showing the percentage of Venus-positive OSNs co-labeled with class I (blue) or class II (magenta) mixed probes (6.58 % in total 1732 Venus+ cells for class I, 0.27 % in total 2190 Venus+ cells for class II probes from 3 Tg mice, $p = 0.00017$, Fisher' s exact test). ****$p < 0.01$. Scale bars, 500 µm.

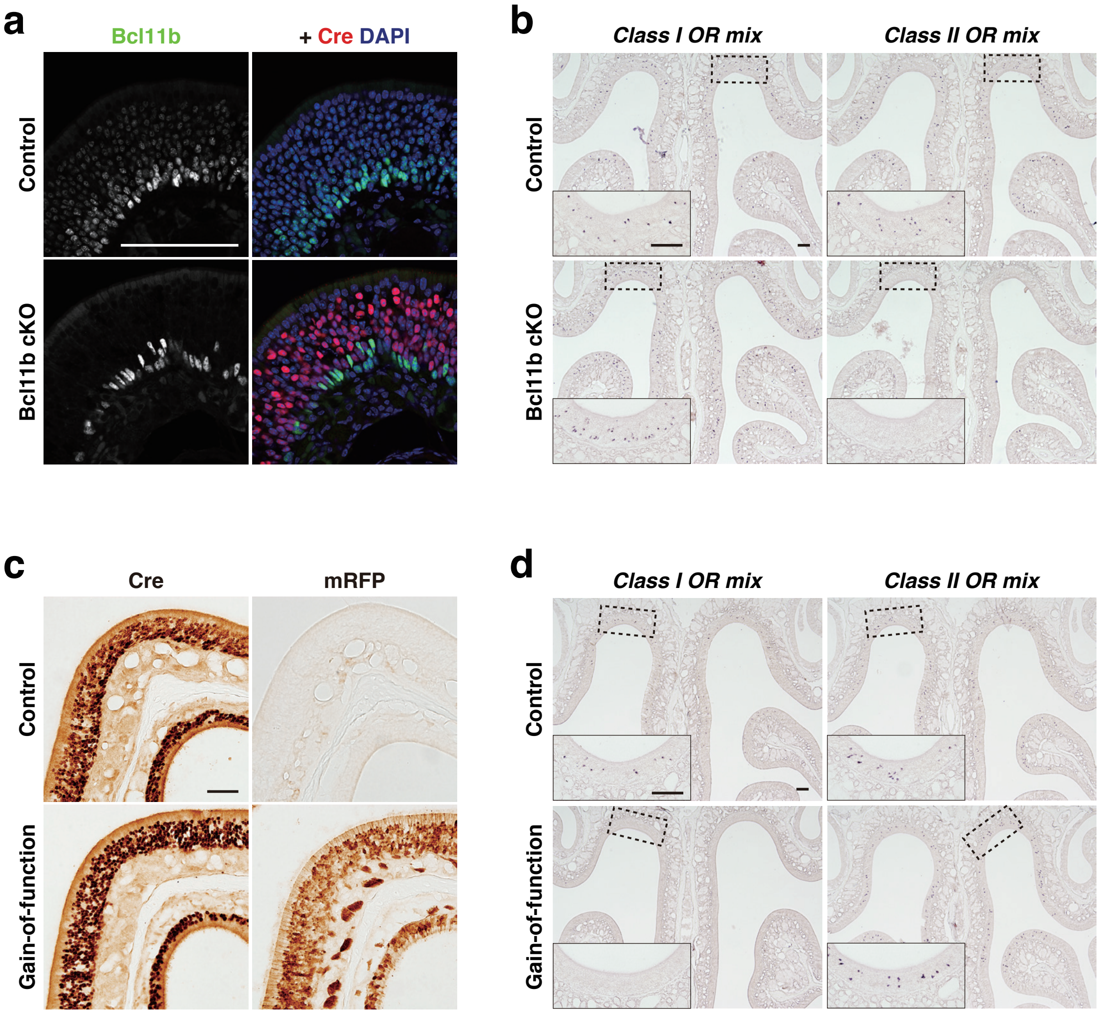

Supplementary Figure 7. Impact of OSN-specific Bcl11b-depletion and -overexpression on the class I and class II OR gene expression.

(**a**) IHC against Bcl11b (green) and Cre (magenta) in the MOE of control and Bcl11b cKO mice at P30. (**b**) ISH with mixed RNA probes for the four class I and the four dorsal class II genes on coronal sections of the MOE of control and Bcl11b cKO mice. (**c**) IHC against Cre and mRFP in the MOE of control and Bcl11b gain-of-function Tg mice. (**d**) ISH with mixed RNA probes for the four class I and the four dorsal class II genes on coronal sections of the MOE of control and Bcl11b gain-of-function Tg mice. Scale bars, 100 µm.

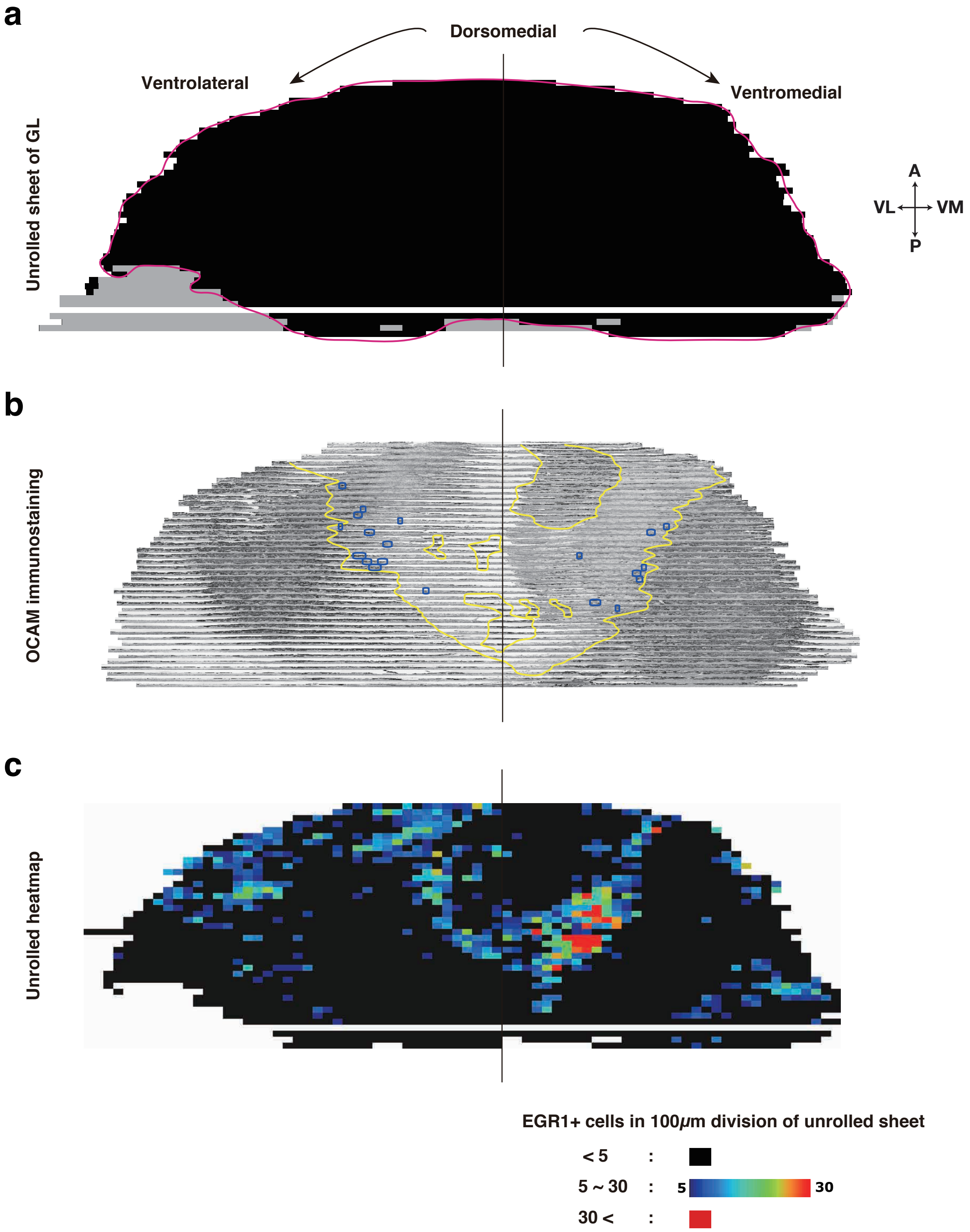

Supplementary Figure 8. Reconstruction of unrolled odor maps.
(**a**) An unrolled sheet of the glomerular layer (GL) of the OB. Vacant areas without glomeruli are shown in gray color. The pink line corresponds to outline of unrolled odor maps. Orientation of unrolled maps is shown by the dorsal-medial edge at the center line to the ventral-medial (VM) side at the right and the ventral-lateral (VL) side at the left. This orientation applies also to Fig. 6. A, anterior; P, posterior. (**b**) An unrolled map for OCAM immunosignal using consecutive sections in **a**. The yellow lines demarcate between OCAM-negative and -positive areas on the unrolled map. The blue circles indicate OCAM-positive glomerulus in OCAM-negative dorsal region of the OB. (**c**) An unrolled heatmap of the OB stimulated with 2MBA. The number of Egr1-positive cells every 100 μm division was indicated by the rainbow color, black to red. Color scale is shown in bottom.

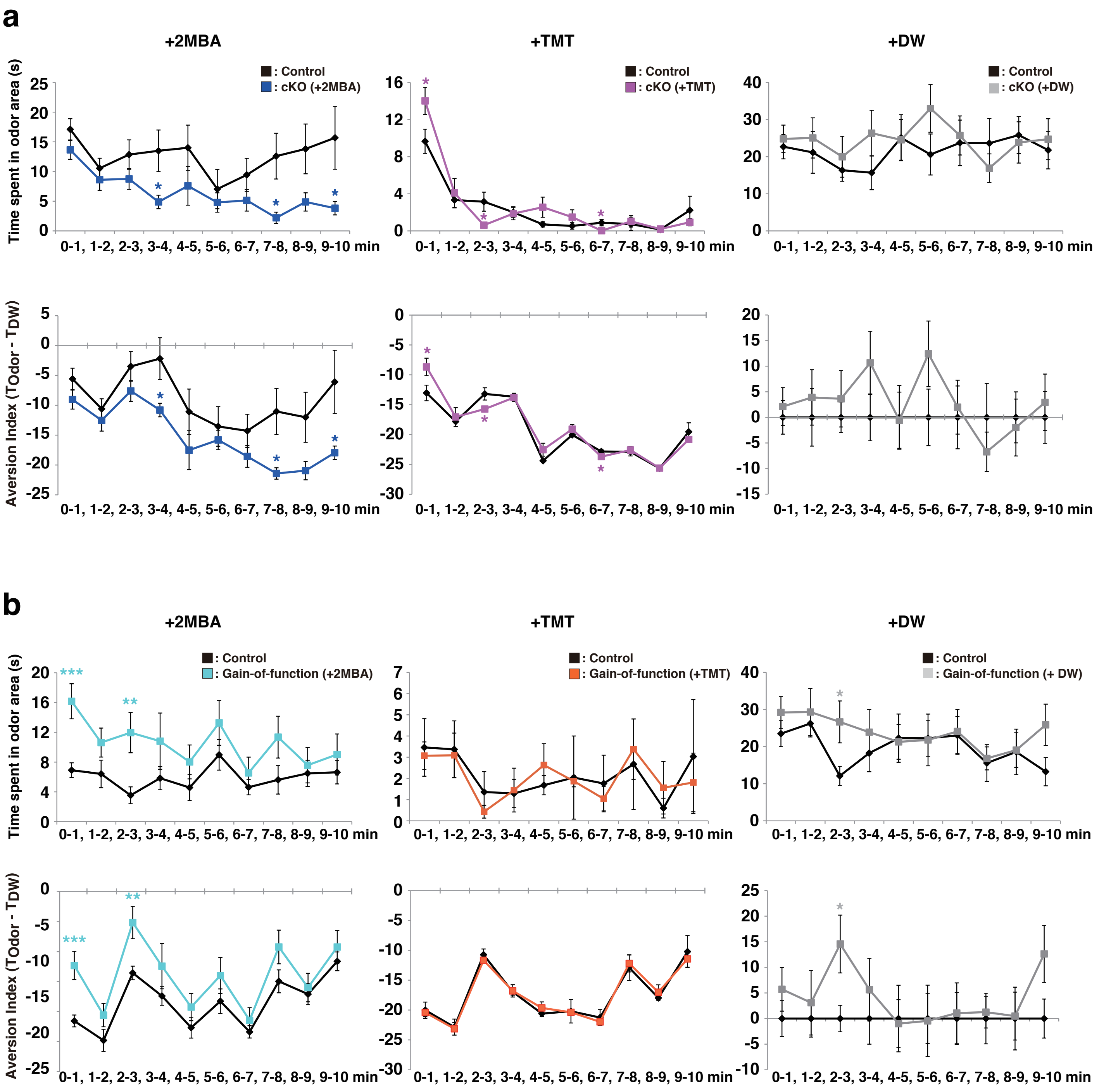

Supplementary Figure 9. Time window of the aversive behaviors in class I- and class II-dominant nose mice, related to Fig. 7.

(**a**) Aversive behaviors to 2MBA (blue for Bcl11b cKO), TMT (magenta for Bcl11b cKO) and DW for control (gray for Bcl11b cKO) in class I-dominant nose mice. Controls are shown by black in each graph. (**b**) Aversive behaviors to 2MBA (cyan for Bcl11b gain-of-function Tg), TMT (orange for Bcl11b gain-of-function Tg) and DW for control (dark gray for Bcl11b gain-of-function Tg) in class II-dominant nose mice. Controls are shown by black in each graph. The graphs show the time a given mouse spent in 1/3 area of odor source (magenta region in Fig. 7a) and aversion index (T_{Odor}-T_{DW}) every 1 min duration. *$p < 0.05$; **$p < 0.01$; ***$p < 0.005$ (two-tailed t-test). All data is summarized in Supplementary Table 1.

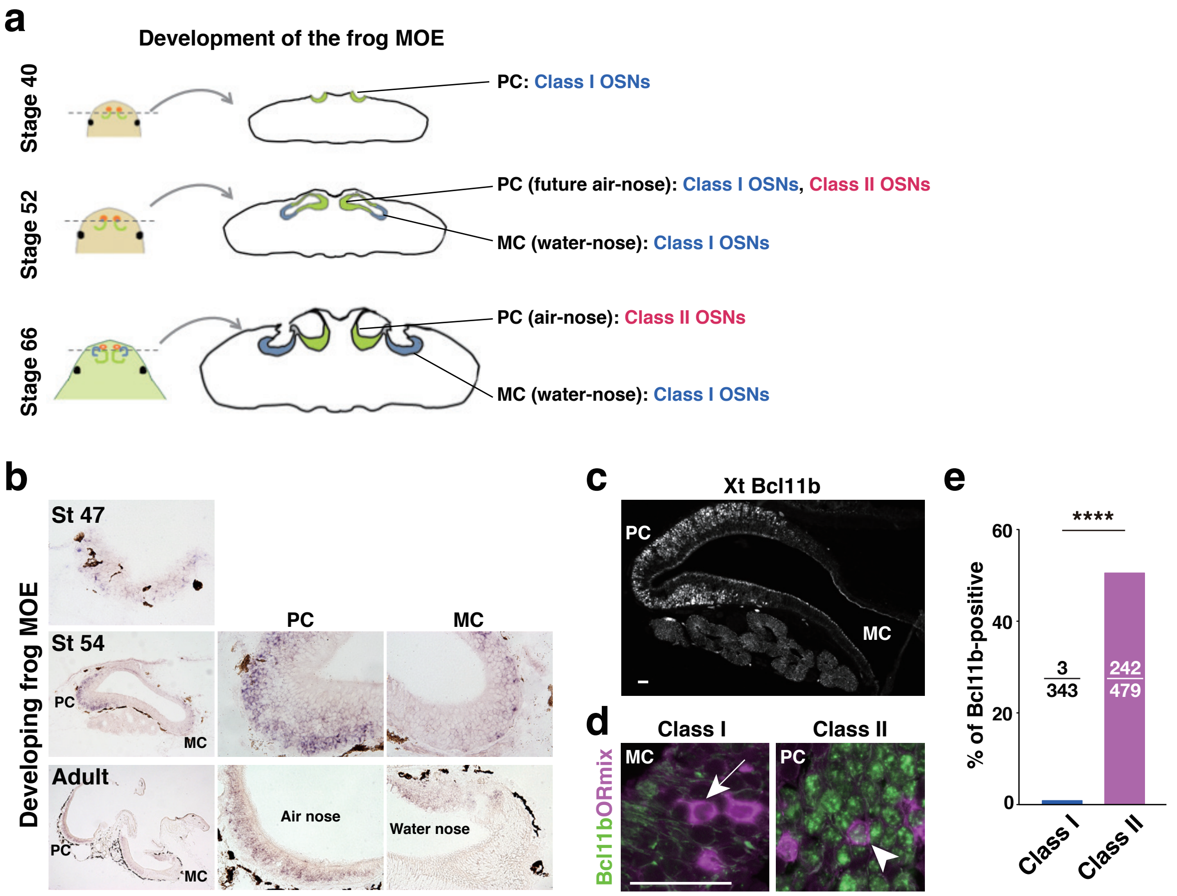

Supplementary Figure 10. The expression profile of Bcl11b in the olfactory epithelium during the tadpole and frog development.

(**a**) Schematic illustration of the OE remodeling and OR expression during the development of frog (*X. tropicalis*). (**b**) ISH with an RNA probe for *XtBcl11b* in the primary olfactory organs of the developing frog (Stage 47, 54 and 60). PC, principal cavity; MC, medium cavity. (**c**, **d**) Combination of IHC for Bcl11b (green) and ISH for mixed OR probes (magenta) in coronal sections of the frog OE at metamorphic stage (stage 56). **c** is low-magnification image of Bcl11b immunoreactivity. **d** is high-magnification images in the MC and PC. Arrowhead and arrow indicate OSN co-labeled and not co-labeled with anti-Bcl11b antibody, respectively. Scale bars 30 µm. (**e**) Percentages of Bcl11b-positive cells that are co-labeled with mixed OR probes for class I (blue) and class II (magenta) (0.87 % in total 479 class I OR-expressing cells, 50.5 % in total 343 class II OR-expressing cells, n = 3, p = 2.2 × 10^-16, Fisher' s exact test). ****$p < 0.001$.
