# Supplementary material for: Bcl11b controls odorant receptor class choice in mice
Source: Commun Biol. 2019 Aug 7;2:296. doi: 10.1038/s42003-019-0536-x (PMC6685970; doi:10.1038/s42003-019-0536-x)
Supplement: Supplementary file 2 — Description of Additional Supplementary Files [file 42003_2019_536_MOESM2_ESM.pdf]

Description of Additional Supplementary Files

The following files are provided as excel files.

Supplementary Data 1. Summary of quantification experiments.
All quantification data, number experiments/animals, and statistical data are summarized.

Supplementary Data 2. Microarray data.
Microarray data of 5 wild type and 6 Bcl11b-/- mice. Probe sets for mature OSN specific genes, OR genes, genes enriched on OR promoters, and genes enriched on OR enhancers are summarized in each sheet.

Supplementary Data 3. RNA-seq FPKMs of OR genes.
RPKM values of each OR gene (gene name, gene ID, chromosome number and position) of control and overexpression mice are summarized.

Supplementary Data 4. List of critical reagents used in this study.
Information of critical reagents used in this study are summarized along with experimental conditions (primary antibodies, riboprobes, PCR primers, mouse lines, software).
