# Supplementary material for: Bcl11b controls odorant receptor class choice in mice
Source: Commun Biol. 2019 Aug 7;2:296. doi: 10.1038/s42003-019-0536-x (PMC6685970; doi:10.1038/s42003-019-0536-x)
Supplement: Supplementary file 7 — Reporting Summary [file 42003_2019_536_MOESM7_ESM.pdf]

nature research

Corresponding author(s): Junji Hirota

Last updated by author(s): Jun 30, 2019

Reporting Summary

Nature Research wishes to improve the reproducibility of the work that we publish. This form provides structure for consistency and transparency in reporting. For further information on Nature Research policies, see Authors & Referees and the Editorial Policy Checklist.

Statistics

For all statistical analyses, confirm that the following items are present in the figure legend, table legend, main text, or Methods section.

n/a	Confirmed	
☐	☒	The exact sample size (*n*) for each experimental group/condition, given as a discrete number and unit of measurement
☐	☒	A statement on whether measurements were taken from distinct samples or whether the same sample was measured repeatedly
☐	☒	The statistical test(s) used AND whether they are one- or two-sided<br>*Only common tests should be described solely by name; describe more complex techniques in the Methods section.*
☒	☐	A description of all covariates tested
☐	☒	A description of any assumptions or corrections, such as tests of normality and adjustment for multiple comparisons
☐	☒	A full description of the statistical parameters including central tendency (e.g. means) or other basic estimates (e.g. regression coefficient) AND variation (e.g. standard deviation) or associated estimates of uncertainty (e.g. confidence intervals)
☐	☒	For null hypothesis testing, the test statistic (e.g. *F*, *t*, *r*) with confidence intervals, effect sizes, degrees of freedom and *P* value noted<br>*Give P values as exact values whenever suitable.*
☒	☐	For Bayesian analysis, information on the choice of priors and Markov chain Monte Carlo settings
☒	☐	For hierarchical and complex designs, identification of the appropriate level for tests and full reporting of outcomes
☒	☐	Estimates of effect sizes (e.g. Cohen's *d*, Pearson's *r*), indicating how they were calculated

Our web collection on statistics for biologists contains articles on many of the points above.

Software and code

Policy information about availability of computer code

Data collection	The source and references of all software were listed in the Supplementary Table 4 and the method section.
Data analysis	Statistical analyses were performed by Microsoft Excel (Version 12.3.6), R (3.3.0) and and GraphPad Prism.

For manuscripts utilizing custom algorithms or software that are central to the research but not yet described in published literature, software must be made available to editors/reviewers. We strongly encourage code deposition in a community repository (e.g. GitHub). See the Nature Research guidelines for submitting code & software for further information.

Data

Policy information about availability of data

All manuscripts must include a data availability statement. This statement should provide the following information, where applicable:

- Accession codes, unique identifiers, or web links for publicly available datasets
- A list of figures that have associated raw data
- A description of any restrictions on data availability

Data availability is stated in the method section.
The raw microarray data and RNA-seq data have been deposited in the Gene Expression Omnibus under ID codes GSE90700 and GSE118169.
The data that support the findings of this study are available from the corresponding author upon reasonable request.

Field-specific reporting

Please select the one below that is the best fit for your research. If you are not sure, read the appropriate sections before making your selection.

☒ Life sciences ☐ Behavioural & social sciences ☐ Ecological, evolutionary & environmental sciences

For a reference copy of the document with all sections, see nature.com/documents/nr-reporting-summary-flat.pdf

Life sciences study design

All studies must disclose on these points even when the disclosure is negative.

Sample size	No statistical methods were used for determining sample sizes. Our sample sizes are similar to those previously reported in the field.
Data exclusions	No data were excluded in our study.
Replication	The number of animals and experiments are listed in the Supplementary Table 1.<br>The number of animals and independently replicated experiments is described in the figure legends.
Randomization	Our data was not analysed with randomization, as similar experiments.
Blinding	Quantification experiments of Fig.1c, Supplementary Fig. 2e, 4c, 4d, 4e, 6d were done blinded to exclude experimenter bias.

Reporting for specific materials, systems and methods

We require information from authors about some types of materials, experimental systems and methods used in many studies. Here, indicate whether each material, system or method listed is relevant to your study. If you are not sure if a list item applies to your research, read the appropriate section before selecting a response.

Materials & experimental systems

n/a	Involved in the study	
☐	☒	Antibodies
☒	☐	Eukaryotic cell lines
☒	☐	Palaeontology
☐	☒	Animals and other organisms
☒	☐	Human research participants
☒	☐	Clinical data

Methods

n/a	Involved in the study	
☒	☐	ChIP-seq
☒	☐	Flow cytometry
☒	☐	MRI-based neuroimaging

Antibodies

Antibodies used	All antibodies used are listed in the Supplementary Table 4.
Validation	Profile data of antibodies provided in each instruction manual and website from manufactures. Manufacture's ID and reference are listed in the Supplementary Table 4.

Animals and other organisms

Policy information about studies involving animals; ARRIVE guidelines recommended for reporting animal research

Laboratory animals	All information of gene targeting mouse strains and transgenic mouse strains is listed in Supplementary Table 4. The conventional Bcl11b knockout mouse strain (Bcl11b-/-) was maintained on a genetic background with BALB/c, other strains were a mixed genetic background. Mutant and wild type mice/embryos of either sex were used for all experiments except physiological and behavioral tests, where only males were used. Data collection of the physiological and behavioral tests occurred from mice >6 weeks of age (details were described in method section).
Wild animals	N/A
Field-collected samples	N/A
Ethics oversight	All animal studies were approved by the Institutional Animal Experiment Committee of the Tokyo Institute of Technology, and were performed in accordance with institutional and governmental guidelines.

Note that full information on the approval of the study protocol must also be provided in the manuscript.
